# Transdiagnostic subgroups of cognitive impairment in early affective and psychotic illness

**DOI:** 10.1038/s41386-023-01729-7

**Published:** 2023-09-22

**Authors:** Julian Wenzel, Luzie Badde, Shalaila S. Haas, Carolina Bonivento, Tamsyn E. Van Rheenen, Linda A. Antonucci, Anne Ruef, Nora Penzel, Marlene Rosen, Theresa Lichtenstein, Paris Alexandros Lalousis, Marco Paolini, Alexandra Stainton, Udo Dannlowski, Georg Romer, Paolo Brambilla, Stephen J. Wood, Rachel Upthegrove, Stefan Borgwardt, Eva Meisenzahl, Raimo K. R. Salokangas, Christos Pantelis, Rebekka Lencer, Alessandro Bertolino, Joseph Kambeitz, Nikolaos Koutsouleris, Dominic B. Dwyer, Lana Kambeitz-Ilankovic, the PRONIA consortium, Mark Sen Dong, Anne Erkens, Eva Gussmann, Shalaila Haas, Alkomiet Hasan, Claudius Hoff, Ifrah Khanyaree, Aylin Melo, Susanna Muckenhuber-Sternbauer, Janis Kohler, Omer Faruk Ozturk, David Popovic, Adrian Rangnick, Sebastian von Saldern, Rachele Sanfelici, Moritz Spangemacher, Ana Tupac, Maria Fernanda Urquijo, Johanna Weiske, Antonia Wosgien, Stephan Ruhrmann, Linda Betz, Karsten Blume, Mauro Seves, Nathalie Kaiser, Tanja Pilgram, Thorsten Lichtenstein, Christiane Woopen, André Schmidt, Anita Riecher-Rössler, Christina Andreou, Laura Egloff, Fabienne Harrisberger, Claudia Lenz, Letizia Leanza, Amatya Mackintosh, Renata Smieskova, Erich Studerus, Anna Walter, Sonja Widmayer, Katharine Chisholm, Chris Day, Sian Lowri Griffiths, Mariam Iqbal, Mirabel Pelton, Pavan Mallikarjun, Ashleigh Lin, Alexander Denissoff, Anu Ellila, Tiina From, Markus Heinimaa, Tuula Ilonen, Paivi Jalo, Heikki Laurikainen, Maarit Lehtinen, Antti Luutonen, Akseli Makela, Janina Paju, Henri Pesonen, Reetta-Liina Armio (Säilä), Elina Sormunen, Anna Toivonen, Otto Turtonen, Jarmo Hietala, Mirka Kolkka, Sinikka Luutonen, Maija Walta, Lauri Tuominen, Ana Beatriz Solana, Manuela Abraham, Nicolas Hehn, Timo Schirmer, Carlo Altamura, Marika Belleri, Francesca Bottinelli, Adele Ferro, Marta Re, Emiliano Monzani, Mauro Percudani, Maurizio Sberna, Armando D’Agostino, Lorenzo Del Fabro, Giampaolo Perna, Maria Nobile, Alessandra Alciati, Matteo Balestrieri, Giuseppe Cabras, Franco Fabbro, Marco Garzitto, Sara Piccin, Giuseppe Blasi, Giulio Pergola, Grazia Caforio, Leonardo Facio, Tiziana Quarto, Barbara Gelao, Raffaella Romano, Ileana Andriola, Andrea Falsetti, Marina Barone, Roberta Passatiore, Marina Sangiuliano, Marian Surman, Olga Bienek, Frauke Schultze-Lutter, Christian Schmidt-Kraepelin, Susanne Neufang, Alexandra Korda, Henrik Rohner

**Affiliations:** 1 Department of Psychiatry and Psychotherapy, Faculty of Medicine and University Hospital, University of Cologne, Cologne, Germany; 2 Department of Psychiatry, Icahn School of Medicine at Mount Sinai, New York, New York, NY USA; 3 Scientific Institute, IRCCS E, Medea, Pasian di Prato, Udine, Italy; 4 Centre for Mental Health, School of Health Sciences, Swinburne University of Technology, Melbourne, VIC Australia; 5 Melbourne Neuropsychiatry Centre, Department of Psychiatry, University of Melbourne & Melbourne Health, Melbourne, VIC Australia; 6 Department of Psychiatry and Psychotherapy, Ludwig-Maximilian University, Munich, Germany; 7 Department of Translational Biomedicine and Neuroscience - University of Bari Aldo Moro, Bari, Italy; 8 Institute of Psychiatry, Psychology & Neuroscience, Department of Psychosis Studies, King’s College London, London, UK; 9 Department of Radiology, University Hospital, Ludwig-Maximilian University, Munich, Germany; 10 Orygen, Melbourne, VIC Australia; 11 Centre for Youth Mental Health, University of Melbourne, Melbourne, VIC Australia; 12 Institute for Translational Psychiatry, University of Muenster, Münster, Germany; 13 Department of Child and Adolescent Psychiatry, University of Münster, Münster, Germany; 14 Department of Neuosciences and Mental Health, Fondazione IRCCS Ca’ Granda Ospedale Maggiore Policlinico, Milan, Italy; 15 Department of Pathophysiology and Mental Health, University of Milan, Milan, Italy; 16 School of Psychology, University of Birmingham, Birmingham, UK; 17 Institute of Mental Health and Centre for Human Brain Health, University of Birmingham, Birmingham, UK; 18 Translational Psychiatry Unit (TPU), Department of Psychiatry and Psychotherapy, University of Luebeck, Luebeck, Germany; 19 Department of Psychiatry and Psychotherapy, Medical Faculty, Heinrich-Heine University, Düsseldorf, Germany; 20 Department of Psychiatry, University of Turku, Turku, Finland; 21 Melbourne Neuropsychiatry Centre, University of Melbourne & Western Health, Melbourne, VIC Australia; 22 Max Planck Institute for Psychiatry, Munich, Germany; 23 Faculty of Psychology and Educational Sciences, Department of Psychology, Ludwig-Maximilian University, Munich, Germany; 24 Department for Psychiatry, Psychotherapy und Psychosomatics, University of Augsburg, Augsburg, Germany; 25 Psychiatric University Hospital, University of Basel, Basel, Switzerland; 26 General Electric Global Research Inc, Munich, Germany; 27 Programma 2000, Niguarda Hospital, Milan, Italy; 28 San Paolo Hospital, Milan, Italy; 29 Department of Medical Area, University of Udine, Udine, Italy; 30 Unit of Psychiatry, Department of Medicine (DAME), University of Udine, 33100 Udine, Italy; 31 Lieber Institute for Brain Development, Johns Hopkins Medical Campus, Baltimore, MD USA; 32 Department of Psychiatry and Behavioral Sciences, Johns Hopkins University School of Medicine, Baltimore, MD USA; 33 Psychiatric Unit - Policlinico di Bari, Bari, Italy; 34 Department of Medicine and Surgery, LUM University, Casamassima, Bari, Italy; 35 Department of Humanities, University of Foggia, Foggia, Italy

**Keywords:** Psychosis, Depression

## Abstract

**Abstract:**

Cognitively impaired and spared patient subgroups were identified in psychosis and depression, and in clinical high-risk for psychosis (CHR). Studies suggest differences in underlying brain structural and functional characteristics. It is unclear whether cognitive subgroups are transdiagnostic phenomena in early stages of psychotic and affective disorder which can be validated on the neural level. Patients with recent-onset psychosis (ROP; *N* = 140; female = 54), recent-onset depression (ROD; *N* = 130; female = 73), CHR (*N* = 128; female = 61) and healthy controls (HC; *N* = 270; female = 165) were recruited through the multi-site study PRONIA. The transdiagnostic sample and individual study groups were clustered into subgroups based on their performance in eight cognitive domains and characterized by gray matter volume (sMRI) and resting-state functional connectivity (rsFC) using support vector machine (SVM) classification. We identified an impaired subgroup (*N*_ROP_ = 79, *N*_ROD_ = 30, *N*_CHR_ = 37) showing cognitive impairment in executive functioning, working memory, processing speed and verbal learning (all *p* < 0.001). A spared subgroup (*N*_ROP_ = 61, *N*_ROD_ = 100, *N*_CHR_ = 91) performed comparable to HC. Single-disease subgroups indicated that cognitive impairment is stronger pronounced in impaired ROP compared to impaired ROD and CHR. Subgroups in ROP and ROD showed specific symptom- and functioning-patterns. rsFC showed superior accuracy compared to sMRI in differentiating transdiagnostic subgroups from HC (BAC_impaired_ = 58.5%; BAC_spared_ = 61.7%, both: *p* < 0.01). Cognitive findings were validated in the PRONIA replication sample (*N* = 409). Individual cognitive subgroups in ROP, ROD and CHR are more informative than transdiagnostic subgroups as they map onto individual cognitive impairment and specific functioning- and symptom-patterns which show limited overlap in sMRI and rsFC.

**Clinical trial registry name:**

German Clinical Trials Register (DRKS). Clinical trial registry URL: https://www.drks.de/drks_web/. Clinical trial registry number: DRKS00005042.

## Introduction

Cogive impairment is common to different psychiatric disorders, in particular depression and psychosis. At the same time these disorders show marked heterogeneity regarding the level of impairment of cognitive performance [1, 2]. Cognitive deficits in processing speed and verbal learning are proposed to have a central role in pathogenesis of psychotic illness [3–5], they appear prior to the first episode of psychosis in individuals at clinical high-risk (CHR) [6] and can also be observed in patients with mood disorders [7, 8]. Recent unsupervised machine learning (ML) studies investigating cognitive deficits in psychosis spectrum disorders, major depression, and bipolar disorder, found subgroups of individuals exhibiting different degrees of cognitive impairment, ranging from cognitively spared to severely impaired profiles [1, 2, 9–14]. This suggests that individuals with different diagnoses might share similar cognitive characteristics.

Mixed samples of patients with schizophrenia, and schizo-affective disorder [15], schizophrenia, and bipolar disorder [16, 17], depression, and bipolar disorder [18, 19], and first episode psychosis, and CHR for psychosis [20], can be subgrouped across diagnoses which supports this notion. In this context neurocognition may serve as an interface across psychiatric diagnoses to identify more homogeneous subgroups that show similarities in clinical symptoms and functioning. Though recent psychopathological models conceptualize cognitive impairment as a transdiagnostic dimension of psychopathology [21], it is unclear to which extent individual diagnoses overlap in severity of cognitive impairment and whether unsupervised ML can identify transdiagnostic subgroups with similar cognitive burden and potentially similar psychopathological pathway.

Neuro-endocrino-immunological alterations early in the development are also suggested to be shared both in depressive [22] and psychotic syndrome [23]. According to the neurodevelopmental hypothesis, particularly in psychosis, pathophysiological processes early in the development affect neuronal circuits which impact cognition and social experiences and eventually increase the vulnerability to the illness. Cognitive subgroups in depression and chronic psychosis are altered in brain structure [15, 24–26] and functional brain connectivity [27, 28], suggesting differences in their underlying neurobiological constitution. Supervised ML has shown sensitivity to identify widespread and interrelated brain patterns, which might be helpful to detect the complex patterns underlying cognitive subgroups [29]. To date, no study has investigated whether transdiagnostic cognitive subgroups in the early stages of the illness map onto underlying structural and functional neurobiological signatures. Findings would further clarify the association between early, potentially premorbid, neurobiological alterations and cognitive dysfunction common across disorders.

We aim to investigate whether cognitive subgroups are shared between patients with recent-onset psychosis (ROP), recent-onset depression (ROD) and those at CHR for psychosis using unsupervised ML. We compare single-disease and transdiagnostic subgroup solutions to determine if the transdiagnostic clustering renders single-disease subgrouping obsolete. We confirm this, if cognitive characteristics of the transdiagnostic subgroups overlap with cognitive characteristics of the subgroups identified in the single-disease clusterings. Individuals assigned to more impaired subgroups should show more pronounced brain structural (sMRI) and resting-state functional MRI alterations relative to HC and spared cognitive subgroups as a consequence of early pathophysiological processes.

## Materials and methods

### Sample

A discovery sample included 465 individuals with psychotic or affective illness or at risk for psychosis and 286 HC, between 15 and 40 years, recruited through the PRONIA study (Personalized Prognostic Tool for Early Psychosis Management, www.pronia.eu; German Clinical Trials Register: DRKS00005042) from seven sites (supplementary material). A replication dataset acquired by the same consortium included 433 patients and 178 HC from the same seven sites (Fig. S1). Written informed consent was obtained from the subjects. Each Local Research Ethics Committee declared their ethical approval for the study [30].

Study group-specific inclusion criteria were used [30] in addition to general inclusion and exclusion criteria (supplementary material). ROP were included in the study, if they fulfilled DSM-IV-TR criteria for a psychotic episode, present in the last three months, lasting longer than one week and with a first onset in the last 24 months. ROD were included, if they fulfilled DSM-IV-TR criteria for a first manifestation of a depressive episode, present in the last 3 months and with onset in the last 24 months. CHR status for psychosis was defined as attenuated psychotic symptoms [31], brief limited intermittent psychotic symptoms, cognitive disturbances [32] or positive family history (1st degree relatives) for psychosis/schizotypal personality disorder according to DSM-IV-TR alongside drop in functioning in the last 6 months. HC volunteers were included, if they did not fulfill any current or past DSM-IV-TR axis I or II diagnosis and/or CHR status for psychosis.

After quality control, the final discovery data set consisted of 668 participants (ROP = 140, ROD = 130, CHR = 128, HC = 270; mean age (yrs; SD) = 25.3 (6.0), females = 353, 52.8%). For the imaging analyses additional 15 discovery participants were excluded due to excessive head movement during the MRI and missing images. The final replication data set consisted of 409 participants (ROP = 108, ROD = 81, CHR = 100, HC = 120; mean age (yrs; SD) = 24.54 (5.7), females = 215, 52.6%; Table 1; Fig. S1; supplementary material).

**Table 1:** Demographic and clinical characteristics of the discovery and replication sample.

	Discovery		Replication		Patient comparison		HC comparison	
					*t*/chi^2^	*p*	*t*/chi^2^	*p*
N	Pat (398)	HC (270)	Pat (289)	HC (120)	-	-	-	-
Age (sd)	25.17 (5.65)	25.40 (6.32)	24.66 (6.05)	24.42 (5.28)	1.1	0.38	1.6	0.22
Site^a^	128/52/66/33/49/42/28	58/39/59/43/23/35/13	137/10/54/18/26/9/35	6/23/11/6/30/35/9	47.45	<0.001	60.08	<0.001
Sex, male/female	210/188	105/165	140/149	54/66	1.25	0.38	1.28	0.38
Studygroup	CHR 128/ ROD 130/ ROP 140	HC 270	CHR 100/ ROD 81/ ROP 108	HC 120	1.7	0.55	-	-
Years of education (sd)	14.18 (3.08)	15.68 (3.22)	13.85 (2.89)	15.17 (3.14)	1.45	0.24	1.47	0.24
Illness duration in days^b^ (sd)	205.26 (188.16)	-	234.52 (214.00)	-	−1.51	0.23	-	-
Chlorpromazine equivalent^c^ (sd)	293.30 (789.28)	-	244.25 (465.73)	-	0.61	0.62	-	-
WAIS Voc (sd)	10.69 (3.13)	11.82 (2.90)	10.43 (3.84)	12.65 (2.54)	0.95	0.45	−2.84	0.02
WAIS Matr (sd)	10.33 (2.51)	11.14 (2.23)	10.49 (3.82)	11.94 (1.97)	−0.63	0.62	−3.56	<0.001
GAF S Lifetime (sd)	80.24 (8.89)	88.04 (5.69)	77.89 (9.14)	87.60 (5.01)	3.33	0.01	0.76	0.55
GAF S Past year (sd)	66.33 (14.66)	86.97 (6.24)	62.17 (13.52)	86.64 (5.59)	3.81	<0.001	0.51	0.65
GAF S Past month (sd)	50.41 (14.00)	86.54 (6.64)	47.14 (13.52)	86.38 (5.60)	3.06	0.01	0.25	0.8
GAF D Lifetime (sd)	79.61 (8.66)	86.73 (5.31)	78.56 (8.74)	87.60 (4.27)	1.55	0.23	−1.72	0.2
GAF D Past year (sd)	67.28 (14.21)	85.64 (6.05)	63.87 (13.79)	86.58 (4.88)	3.13	0.01	−1.63	0.22
GAF D Past month (sd)	52.19 (14.51)	85.14 (6.41)	48.63 (13.91)	86.38 (4.83)	3.22	0.01	−2.09	0.1
PANSS Pos (sd)	13.03 (6.12)	-	14.16 (6.39)	-	−2.29	0.07	-	-
PANSS Neg (sd)	13.85 (6.60)	-	14.75 (6.55)	-	−1.75	0.2	-	-
PANSS Gen (sd)	30.38 (9.13)	-	31.94 (9.02)	-	−2.17	0.09	-	-
BDI (sd)	22.60 (12.07)	3.25 (4.74)	23.01 (12.16)	3.57 (5.55)	−0.41	0.7	−0.52	0.65

^a^institute IDs in this order: LMU/BAS/UKK/BHAM/TUR/UD/MIL.

^b^calculated only for ROP, ROD.

^c^cumulative sum of chlorpromazine equivalent divided by number of days treated; calculated only for ROP.

*WAIS* Wechsler Adult Intelligence Scale, *Voc* Vocabulary subtest, *Matr* Matrix substest, *GAF* Global Assessment of Functioning: symptoms scale, disability scale, *PANSS* Positive and Negative Syndrom Scale: positive, negative and general symptom scale, *BDI* Beck’s Depression Inventory.

### Clinical and cognitive assessment

We assessed clinical symptoms and functioning using the General Assessment of Functioning Scale (GAF) split into a ‘disability’ (D) and ‘symptom’ (S) score [33], the Positive and Negative Syndrome Scale (PANSS) [34] and the Beck Depression Inventory (BDI) [35].

We characterized cognitive performance using a battery of cognitive tests spanning eight cognitive domains according to the MATRICS Consensus Cognitive Battery (MCCB) [36, 37]: visual memory (Rey-Osterrieth Complex Figure Test [38, 39]), social cognition (Diagnostic Analysis of Nonverbal Accuracy [40]), working memory (auditory digit span [41]; self-ordered pointing task [42]), processing speed (verbal fluency test [43]; Trail Making Test A [44]; Digit Symbol Substitution Test [41]), verbal learning and memory (Rey Auditory Verbal Learning Test [45]), executive functioning (Trail Making Test B [44]), attention/vigilance (Continuous Performance Test-Identical Pairs [46]) and salience [47] (Table S1, S2). We assessed intelligence using proxies from the Wechsler Adult Intelligence Scale (WAIS-IV), the vocabulary subtest, and the matrix subtest [41].

### Preprocessing of cognitive variables

Preprocessing and clustering analysis of cognitive variables followed a similar pipeline as recently established [14] (supplementary material). After quality control the final analysis data set consisted of 84 cognitive variables showing on average 0.6% missing values (SD cognitive variables = 0.3%; SD participant = 5.3%). We retained the identical set of cognitive variables in the replication data where preprocessing and statistical analysis followed the same steps (Fig. S1). The final replication data set showed on average 0.6% missing values (SD_cognitive variables_ = 0.8%; SD_participant_ = 2.4%) prior to the statistical analysis (Fig. S2).

### Dimensionality reduction and K-means clustering analyses

Identical pipelines were conducted for the transdiagnostic clustering and the individual study group clustering and contained the following steps. Cognitive data were imputed and effects of age, sex, years of education, and study site were regressed out. To reduce the dimensionality of the cognitive features, we conducted cognitive domain-wise principal component analyses (PCA) (Table S2; Fig. S3) retaining the first component of each cognitive domain (*N* = 8). The eight cognitive domain scores were used in a K-means clustering analysis embedded in a resampling procedure to determine the optimal number of clusters and cluster stability (supplementary material). HC were used as a comparison group to the obtained cognitive subgroups and not part of the clustering procedure.

### Statistical analyses for cluster characterization

We calculated one-factorial Analyses of Variance (ANOVAs) with the factor ‘cluster + HC’ to characterize cognitive, demographic, clinical and functioning differences between clusters and HC. To characterize cognitive differences between subgroups of individual clusterings (ROP, ROD, CHR), we calculated two-factorial ANOVAs with ‘cluster’ and ‘study group’ as between-factors. *P*-values were Benjamini-Hochberg false discovery rate (FDR) corrected [48] within their domain (cognitive, demographic and clinical/functioning) and FDR corrected pairwise *t* tests/chi-squared tests (for nominal scales) were calculated for individual comparisons.

Analyses were conducted in R version 3.6.1 (https://cran.r-project.org/bin/windows/base/). We used the ‘clusterboot’- [49] and ‘kmeansruns’-function contained in the ‘fpc’ package [50] for cluster stability assessment and cluster number estimation.

### Preprocessing of neuroimaging data

Preprocessing and quality control of the gray matter (GM) images followed the protocols established previously [30] and the CAT12 manual (www.neuro.uni-jena.de/cat12/CAT12-Manual.pdf), respectively. The rsfMRI data preprocessing followed the protocol established in ref. [51]. In brief, rsfMRI data were parcellated into 160 regions of interest according to the Dosenbach functional atlas [52] and mean signal was extracted from 10 mm spheres around each region. We calculated pairwise Pearson’s correlations of the average time series between ROIs using in-house scripts running in Matlab R2015 resulting in connectivity matrices of 12720 resting-state functional connectivity (rsFC) features per participant (supplementary material).

### Neuroimaging classification analyses

We built supervised ML models to assess the discriminability of the transdiagnostic clusters with respect to the GM volume and rsFC brain features. Classification performance between the obtained cognitive subgroups was assessed in two separate supervised ML pipelines each embedded in 10 × 10 repeated nested cross-validation using NeuroMiner (http://www.pronia.eu/neurominer) running in a MATLAB 2019a environment (MathWorks Inc.). We used an optimized linear support vector machine (SVM) algorithm and assessed classification performance based on balanced accuracy (BAC). We applied permutation testing (*N*_perm_ = 100; alpha = 0.05) to assess the final model significance (supplementary material). Using the same pipelines, we conducted classification analyses between individual study groups and HC, e.g., all ROP individuals against HC, to investigate whether the transdiagnostic subgrouping increased prediction accuracy relative to the individual study group classification.

### External validation analyses

We projected the centroids of the transdiagnostic discovery sample cluster solution into the data spaces of the transdiagnostic replication sample. Similarly, we project the centroids of the individual study group cluster solutions into the data spaces of the respective study groups of the replication sample. Participants were assigned to the closest cluster centroid using Euclidean distance. We evaluated the validity of the external replication of the clusters relative to the effects obtained for the discovery solution (supplementary material).

## Results

We identified a highly stable two cluster-solution (supplementary material, Fig. S4). Cluster 1 (*N* = 146) consisted of 79 (54%) ROP, 30 (21%) ROD and 37 (25%) CHR. Cluster 2 (*N* = 252) consisted of 61 (21%) ROP, 100 (38%) ROD and 91 (41%) CHR. We obtained a higher proportion of ROD in cluster 2 (*p* < 0.001) and a higher proportion of ROP in cluster 1 (X^2^(2, 398) = 37.195, both *p* < 0.001; table S3).

### Differences in cognitive performance between transdiagnostic clusters

**Fig. 1:**
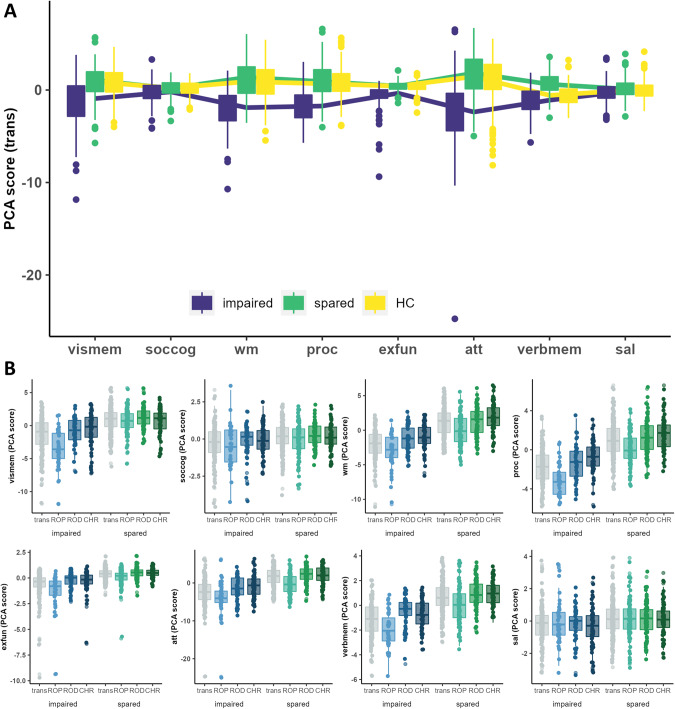
Cognitive characteristics of clusters based on the transdiagnostic and individual clustering analyses in the discovery sample. **A** represents the cognitive performances of impaired (blue) and spared clusters (green) for the transdiagnostic cluster solution. **B** represents the cognitive performances of impaired (shades of blue) and spared (shades of green) clusters for the clusterings based on recent-onset depression patients (ROD), recent-onset psychosis patients (ROP) and clinical high-risk individuals (CHR) separately. For comparison impaired and spared clusters of the transdiagnostic cluster solution are shown in gray. For both sections: High principal component (PCA) scores represent high performance. Abbreviations: vismem visual memory, soccog social cognition, wm working memory, proc processing speed, exfun executive functioning, att attention, sal salience, verbmem verbal memory.

Analyses revealed reduced cognitive performance in cluster 1 when compared to cluster 2 across all cognitive domains, i.e., social cognition, working memory, processing speed, executive functioning, attention, visual memory, verbal memory and salience (main effect: cluster 1/cluster 2/HC: F(2,665) > 5.822, *p* < 0.01; individual cluster comparisons: *p* < 0.001 for all cognitive domains except for salience: *p* < 0.01). Whereas cluster 1 performed significantly worse than HC across all cognitive domains (*p* < 0.01) except for salience (*p* = 0.110), cluster 2 performed comparable to HC with respect to social cognition (*p* = 0.920), processing speed (*p* = 0.170), executive functioning (*p* = 0.370), verbal memory (*p* = 0.71), attention (*p* = 0.130), visual memory (*p* = 0.640) and salience (*p* = 0.056). Additionally, cluster 2 performed better than HC with respect to working memory (*p* < 0.05). We found the same pattern for the WAIS vocabulary and matrix scores which were not part of the clustering procedure (Table 2; Fig. 1).

**Table 2:** Cognitive characteristics of the transdiagnostic cluster solution in discovery and replication sample.

	Transdiagnostic					
	Impaired	Spared	HC	ANOVA		
				*F*	*p* fdr	Individual cluster comparisons
Discovery sample						
* N*	146	252	270			
soccog	−0.28 (1.14)	0.16 (0.87)	0.17 (0.74)	14.23	<0.001	imp < sp; imp < HC; sp = HC
wm	−1.98 (2.08)	1.15 (2.04)	0.79 (1.97)	123.11	<0.001	imp < sp; sp > HC; imp < HC
proc	−1.74 (1.85)	1.01 (1.76)	0.81 (1.52)	141.63	<0.001	imp < sp; imp < HC; sp = HC
exfun	−0.66 (1.3)	0.38 (0.46)	0.32 (0.48)	106.82	<0.001	imp < sp; imp < HC; sp = HC
att	−2.56 (3.55)	1.48 (2.46)	1.12 (2.42)	115.87	<0.001	imp < sp; imp < HC; sp = HC
verbmem	−1.15 (1.43)	0.67 (1.16)	0.63 (1.09)	128.5	<0.001	imp < sp; imp < HC; sp = HC
vismem	−1.39 (2.72)	0.81 (1.8)	0.73 (1.59)	68.81	<0.001	imp < sp; imp < HC; sp = HC
sal	−0.23 (1.14)	0.13 (1.05)	−0.06 (0.95)	5.82	0.003	imp < sp; imp = HC; sp = HC
WAIS						
vocab	9.26 (3.1)	11.52 (2.83)	11.82 (2.9)	39.68	<0.001	imp < sp; imp < HC; sp = HC
matrix	9.09 (2.55)	11.05 (2.18)	11.14 (2.23)	44.15	<0.001	imp < sp; imp < HC; sp = HC
Replication sample						
* N*	161	128	120			
soccog	−0.61 (1.82)	0.73 (1.96)	0.17 (0.81)	24.17	<0.001	imp < sp; sp > HC; imp < HC
wm	−1.72 (2.5)	1.64 (2.72)	0.79 (1.39)	83.71	<0.001	imp < sp; sp > HC; imp < HC
proc	−2.78 (3.1)	0.47 (2.73)	0.81 (1.4)	85.62	<0.001	imp < sp; sp = HC; imp < HC
exfun	−0.72 (1.18)	0.2 (0.9)	0.32 (0.44)	55.28	<0.001	imp < sp; sp = HC; imp < HC
att	−4.85 (6.26)	2.12 (3.58)	1.12 (2.02)	100.87	<0.001	imp < sp; sp = HC; imp < HC
verbmem	−0.39 (1.58)	1.04 (1.32)	0.63 (1)	43.16	<0.001	imp < sp; sp > HC; imp < HC
vismem	−1.62 (3.37)	0.91 (2.17)	0.73 (1.55)	43.84	<0.001	imp < sp; sp = HC; imp < HC
sal	−0.67 (1.6)	−0.76 (2.13)	−0.06 (0.85)	6.99	0.001	sp < imp; imp < HC; sp < HC
WAIS						
vocab	9.72 (3.13)	11.32 (4.43)	12.65 (2.54)	25.24	<0.001	imp < sp; sp < HC; imp < HC
matrix	9.7 (2.67)	11.49 (4.72)	11.94 (1.97)	18.62	<0.001	imp < sp; sp < HC; imp < HC

*soccog* social cognition, *wm* working memory, *proc* processing speed, *exfun* executive functioning, *att* attention, *verbmem* verbal memory, *vismem* visual memory, *sal* salience, *WAIS* Wechsler Adult Intelligence Scale, *vocab* vocabulary subtest, *matrix* matrix subtest, *fdr* false-discovery rate correction, *HC* healthy controls, *sp* spared, *imp* impaired.

Hereafter, cluster 1 is referred to as the impaired cluster, and cluster 2 as the spared cluster.

### Differences in clinical and functioning characteristics between transdiagnostic clusters

**Fig. 2:**
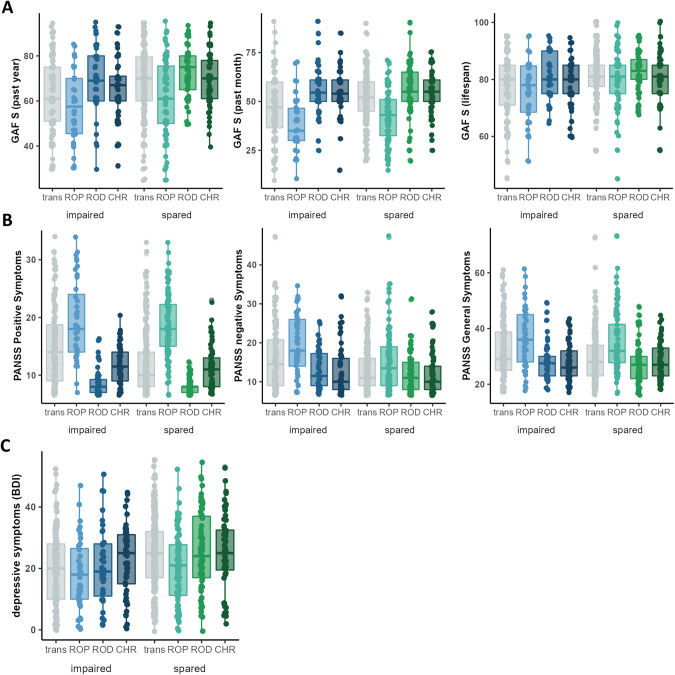
Functional characteristics and characteristics with respect to symptoms of the clusters based on the transdiagnostic and individual clustering analyses in the discovery sample. Functioning differences (**A**) and psychotic and depressive symptom differences (**B**, **C**) of impaired (shades of blue) and spared (shades of green) clusters for the clusterings based on recent-onset depression patients (ROD), recent-onset psychosis patients (ROP) and clinical high-risk individuals (CHR) separately. For comparison impaired and spared clusters of the transdiagnostic cluster solution are shown in gray. Abbreviations: GAF S global assessment of functioning (symptom scale), PANSS positive and negative syndrome scale, BDI Beck’s depression inventory.

The impaired cluster in comparison to the spared cluster showed significantly lower functioning with respect to the GAF symptom scale in the last month (main effect: cluster 1/cluster 2/HC: F(2, 660) = 810.080, *p* < 0.001; impaired vs spared: *p* < 0.001) and the last year (main effect: cluster 1/cluster 2/HC: F(2, 660) = 253.120, *p* < 0.001; impaired vs spared: *p* < 0.001) before study entry as well as across the lifespan (main effect: cluster 1/cluster 2/HC: F(2, 660) = 92.854, *p* < 0.001; impaired vs spared: *p* < 0.001). Both impaired and spared cluster show significantly lower functioning with respect to the GAF symptom scale in the last month (impaired: *p* < 0.001, spared: *p* < 0.001) and the last year (impaired: *p* < 0.001, spared: *p* < 0.001) before study entry as well as across the lifespan (impaired: *p* < 0.001, spared: *p* < 0.001) when compared to HC. Effects between clusters and HC are similar with respect to the GAF disability scale (Table S3; Fig. 2).

The impaired cluster in comparison to the spared cluster showed significantly higher positive (*t*(245.28) = 4.728, *p* < 0.001), negative (*t*(213.82) = 3.955, *p* < 0.001) and general (*t*(245.56) = 2.585, *p* < 0.05) symptoms on the PANSS scale. Both impaired and spared cluster showed significantly higher depressive symptoms on the BDI as compared to HC (main effect: cluster 1/cluster 2/HC: *F*(2, 621) = 323.526, *p* < 0.001; impaired vs HC: *p* < 0.001; spared vs HC: *p* < 0.001) while the spared subgroup showed higher depressive symptoms in comparison to the impaired subgroup (*p* < 0.001).

### Differences in cognitive performance between single-disease clusterings

Similar to the transdiagnostic cluster solution, the individual clusterings (ROP: impaired: *N* = 42 [30%], spared: *N* = 98 [70%]; ROD: impaired: *N* = 45 [35%], spared: *N* = 85 [65%]; CHR: impaired: *N* = 59 [46%], spared: *N* = 69 [54%]) showed an impaired subgroup with widespread reductions in cognitive performance relative to HC and a spared subgroup often performing similar to or better than HC (Supplementary material, Tables S4–S6).

When comparing impaired and spared subgroups across clusterings, we found that the impaired and spared subgroup of the ROP clustering performed significantly worse in comparison to the impaired and spared subgroups of ROD and CHR in the domains of working memory (main effect study group: *F*(2, 392) = 12.687, *p* < 0.001; impaired ROD: *p* < 0.001; impaired CHR: *p* < 0.001), processing speed (main effect study group: *F*(2, 392) = 26.603, *p* < 0.001; impaired ROD: *p* < 0.001; impaired CHR: *p* < 0.001), attention (main effect study group: *F*(2, 392) = 16.453, *p* < 0.001; impaired ROD: *p* < 0.001; impaired CHR: *p* < 0.001), and verbal memory (main effect study group: *F*(2, 392) = 19.371, *p* < 0.001; impaired ROD: *p* < 0.001; impaired CHR: *p* < 0.001). Additionally, we obtained a significant interaction for visual memory (interaction effect: *F*(2, 392) = 14.324, *p* < 0.001) showing that the impaired ROP group performed significantly worse as compared to impaired ROD (*p* < 0.001) and CHR (*p* < 0.001) whereas the spared ROP group performed comparable to the spared ROD (*p* = 0.058) and CHR group (*p* = 0.652). We obtained a significant interaction for executive functioning (interaction effect: *F*(2, 392) = 19.273, *p* < 0.001; impaired ROD: *p* < 0.001; impaired CHR: *p* < 0.001) showing that the impaired ROP group performed significantly worse as compared to impaired ROD and CHR (*p* < 0.001) whereas reductions between the spared ROP group and the spared ROD (*p* < 0.05) and CHR group (*p* < 0.05) were less pronounced (Table S7).

### Differences in clinical and functional characteristics between single-disease clusterings

Impaired and spared cognitive subgroups of the individual clusterings were less distinct with respect to functional impairments (Tables S8–S10, Fig. 2). Whereas impaired and spared subgroups in ROP follow a similar pattern than the transdiagnostic cluster solution, i.e., higher functional impairment in the cognitively impaired in comparison to the cognitively spared subgroup, impaired ROD subgroups and impaired CHR subgroups show less functional impairments. Additionally, we find significantly higher negative symptoms for impaired as compared to spared ROP (*t*(72.823) = 3.006, *p* < 0.01) as well as significantly higher depressive symptoms for spared ROD as compared to impaired ROD and HC (main effect: cluster 1/cluster 2/HC: *F*(2, 377) = 281.619, *p* < 0.001; spared vs impaired: *p* < 0.01; spared vs HC: *p* < 0.001).

### Differences in GM volume between transdiagnostic clusters

**Fig. 3:**
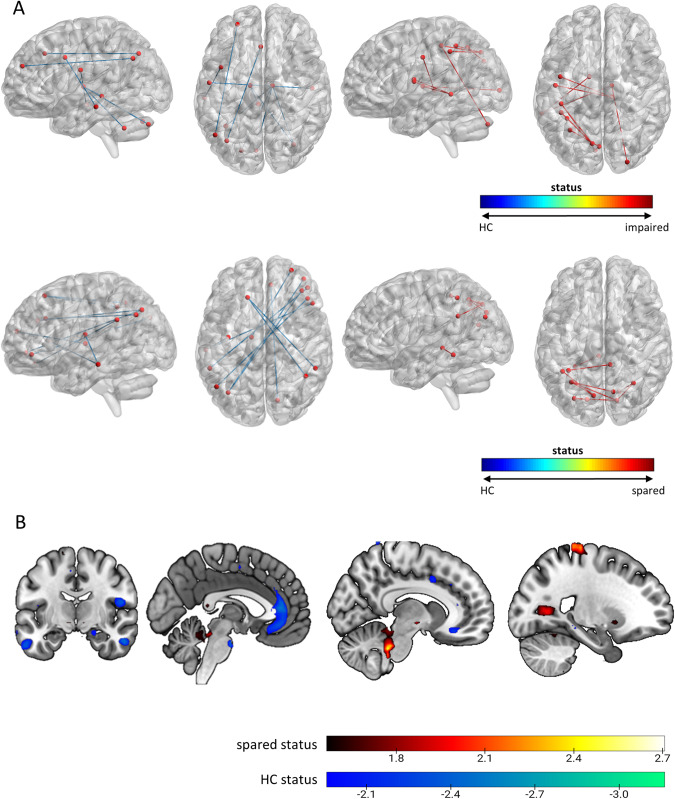
Reliability maps of significant sMRI and rsFC classification models as measured by the cross-validation ratio (CV ratio). The upper row in panel (**A**) shows the predictive connectivity patterns for the ‘impaired vs HC’ rsFC model. The lower row in (**A**) shows the predictive connectivity patterns for the ‘spared vs HC’ rsFc model. The ten most predictive connectivity patterns for HC status are marked in blue and the ten most predictive connectivity patterns for impaired/spared cluster status are marked in red. A list containing the predictive features is given in supplementary Table S12. Figures were generated using the BrainNet Viewer. **B** shows the voxel reliability maps for the significant ‘spared vs HC’ sMRI model. Voxels predictive of spared status are represented by positive CV ratio ( = warm colors) and voxels predictive of HC status are represented by negative CV ratio ( = cool colors). Reliability maps are thresholded at the 99th percentile for both positive and negative CV ratio.

The SVM classification model based on GM volume separated the cognitively spared cluster from HC (BAC = 53.1%, Sensitivity (Sens) = 55.6%, Specificity (Spec) = 50.6%, positive predictive value (PPV) = 51.7%, negative predictive value (NPV) = 54.5%; *p* = 0.04) while it could neither separate the cognitively impaired cluster from HC (BAC = 51.4, Sens = 47.9%, Spec = 54.8%, PPV = 36.9%, NPV = 65.6%; *p* = 0.37) nor the cognitively impaired cluster from the cognitively spared cluster (BAC = 53.0, Sens = 42.4%, Spec = 63.7%, PPV = 40.4%, NPV = 65.6%; *p* = 0.14) (Figs. 3, S5). Classification of the transdiagnostic subgroups provided no or no substantial gain in accuracy over the classification of individual study groups from HC (Table S11).

### Differences in functional connectivity between transdiagnostic clusters

The SVM classification model based on rsFC separated the cognitively impaired cluster (BAC = 58.5, Sens = 73.6%, Spec = 43.3%, PPV = 41.7%, NPV = 74.8%; *p* < 0.01) and cognitively spared cluster (BAC = 61.7, Sens = 62.5%, Spec = 60.9%, PPV = 60.3%, NPV = 63.1%; *p* < 0.01) from HC. The model classifying cognitively spared and impaired cluster was not significant (BAC = 55.9, Sens = 49.3%, Spec = 62.5%, PPV = 43.3%, NPV = 68.0%; *p* > 0.05) (Table S12, Figs. 3, S5). Classification of the transdiagnostic subgroups provided no or no substantial gain in accuracy over the classification of individual study groups from HC (Table S11).

## External validation

### Differences in cognitive, functional and clinical characteristics between transdiagnostic clusters

Similar to the findings in the discovery sample, the transdiagnostic cluster solution of the replication sample showed an impaired subgroup with widespread reductions in cognitive performance relative to HC and a spared subgroup often performing similar to or better than HC (Table 2, Fig. S6).

Transdiagnostic cluster effects with respect to functioning were less pronounced in the replication sample (Table S13, Fig. S7). The impaired cluster in comparison to the spared cluster showed significantly higher positive (*t*(270.63) = 4.603, *p* < 0.001) symptoms on the PANSS scale. The spared subgroup showed higher depressive symptoms in comparison to the impaired subgroup (main effect: cluster 1/cluster 2/HC: *F*(2, 346) = 128.659, *p* < 0.001; spared vs impaired: *p* < 0.05).

### Differences in cognitive, functioning, and clinical characteristics between single-disease clusters

Individual clusterings showed an impaired subgroup with widespread reductions in cognitive performance relative to HC and a spared subgroup often performing comparable to or better than HC. When comparing impaired and spared subgroups across clusterings, we found that the impaired and spared subgroup of the ROP clustering performed significantly worse in comparison to the impaired and spared subgroups of ROD and CHR (Tables S7, S14–S16, Fig. S6).

Impaired ROP, ROD and CHR subgroups did not show significantly more functional impairment than their spared subgroups. Impaired and spared clusters were not distinct with respect to BDI and symptoms on the PANSS (Tables S17–S19, Fig. S7).

## Discussion

The current study identified spared and impaired cognitive subgroups across a transdiagnostic sample and in patients with affective and psychotic illness and CHR state. Impaired subgroups showed widespread cognitive impairment while the spared subgroups showed cognitive performance comparable to HC. Single-disease clustering analyses indicated that ROP were characterized by more impairment in both impaired and spared subgroups than the ROD and CHR groups and provided a more refined picture on functional impairments and symptoms associated with cognitive subgroups than the transdiagnostic clustering solution. We found a higher discriminability of the transdiagnostic cognitive subgroups based on rsfMRI than on sMRI. Analyses based on rsfMRI showed that transdiagnostic clusters were significantly differentiated from HC.

Previous studies of patients with established psychiatric illness and at CHR identified subgroups showing a ‘severe cognitive deficit’ as well as a subgroup with ‘preserved cognitive performance’ [1, 2, 9, 10, 13–16, 18–20]. We showed that this cognitive heterogeneity is also present at an early stage in psychotic and depressive disorders and at CHR. Cognitive impairments in impaired subgroups were most strongly pronounced in working memory, verbal memory, processing speed and attention. Consistently, metanalytical findings show that processing speed, verbal memory and working memory count among the most strongly impaired cognitive domains in schizophrenia and depression [21, 53]. As evident from single-disease clustering, ROP showed significantly more impairment with respect to these cognitive domains than impaired ROD or CHR. Further, processing speed and verbal memory represent the most predictive cognitive domains for transition to psychosis in CHR [54]. Our findings indicate that though cognitive impairment is a transdiagnostic phenomenon which shows substantial heterogeneity, individuals with impaired cognition in psychosis, depression and CHR state vary in severity [21]. To explain these variations differences in pharmacological treatment [21] as well as in illness duration [55] might play a limited role in the current study as individuals showed only short-term exposure to pharmacological treatment and ROP showed shorter illness duration than ROD. Therefore, illness-specific psychopathological characteristics have likely contributed to the differences [21].

Cognitively impaired subgroups across individual psychiatric conditions have been associated with greater deficits in functioning [20, 56–62] and higher burden in clinical symptoms [57, 59, 61]. The impaired transdiagnostic subgroup was associated with more functional impairment compared to the transdiagnostic spared subgroup and HC. Consistent with our cognitive findings in the single-disease clustering, we found that functional impairment in the cognitively impaired subgroup is more pronounced in ROP than in ROD and CHR. The transdiagnostic clustering indicated significantly higher positive, negative, and general symptoms in the impaired subgroup as compared to the spared subgroup and HC. The effect for negative symptoms seemed to be driven by the cognitively impaired subgroup in ROP which is consistent with previous literature [61, 62]. The spared transdiagnostic subgroup showed significantly higher depressive symptoms as compared to the impaired subgroup and HC which seemed to be mainly driven by spared ROD. Metanalyses in depression show inconsistent findings for the association of cognition and depressive symptom severity [63–65].

Both GMV and functional connectivity alterations have been identified in schizophrenia, CHR and depression [66–68]. SVM classifiers based on rsFC showed superior accuracy in differentiating the impaired and spared transdiagnostic subgroups which is in line with findings reporting higher sensitivity of ML algorithms in the functional resting-state compared to the neuroanatomical brain modality [29]. Transdiagnostic spared and impaired clusters were classified with relatively low accuracy in both imaging modalities. We found no or no substantial gains in classification accuracy when classifying the transdiagnostic subgroups as compared to individual study groups against HC. In sum, this suggests that transdiagnostic cognitive impairment in early illness accounts for a small amount of variance in structural and resting-state functional brain measurements.

We validated the discovery sample results regarding the transdiagnostic spared and impaired subgroup in the PRONIA replication sample and confirmed the finding that cognitive dysfunction in the impaired cognitive subgroups is more pronounced in ROP as compared to ROD and CHR. Cognitive subgroups in the replication sample were less associated with differences in general functioning and symptoms.

There are limitations to our findings. First, different numbers of cognitive variables per cognitive domain were used for clustering leading to an underrepresentation of certain cognitive domains, e.g., in social cognition (Table S2) [14]. Second, we used SVM algorithms due to their high interpretability while non-linear classification algorithms, such as deep neural networks [69] might have revealed higher classification accuracies. Further, focusing the analyses on apriori defined brain networks [70] might have increased the sensitivity to differentiate the groups. Third, classification performance in the imaging analyses might have been limited due to differences in sample size between groups though we weighted the hyperplane of the SVM algorithm in favor of the minority group. Fourth, due to low SVM classification accuracies in the discovery sample we did not apply our brain models to the replication sample.

We provide evidence that ROP, ROD and CHR differ in cognitive heterogeneity while cognitive subgroups in individual study groups map onto different general functioning and symptoms characteristics. Transdiagnostic impaired subgroups did not reveal increased classification performance in the investigated structural and functional brain modalities relative to the spared subgroup and HC, indicating heterogeneity in underlying neurobiological patterns for the identified transdiagnostic subgroups. Study group specific cognitive subgroups might be more informative than transdiagnostic subgroups.

### Supplementary information

Supplementary Material

Table S1

## References

[1] Green MJ, Girshkin L, Kremerskothen K, Watkeys O, Quidé Y (2020). A systematic review of studies reporting data-driven cognitive subtypes across the psychosis spectrum. Neuropsychol Rev.

[2] Pu S, Noda T, Setoyama S, Nakagome K (2018). Empirical evidence for discrete neurocognitive subgroups in patients with non-psychotic major depressive disorder: clinical implications. Psychol Med.

[3] Sheffield JM, Karcher NR, Barch DM (2018). Cognitive deficits in psychotic disorders: a lifespan perspective. Neuropsychol Rev.

[4] Kahn RS, Keefe RSE (2013). Schizophrenia is a cognitive illness: time for a change in focus. JAMA Psychiatry.

[5] Kremen WS, Seidman LJ, Faraone SV, Toomey R, Tsuang MT (2000). The paradox of normal neuropsychological function in schizophrenia. J Abnorm Psychol.

[6] Fusar-Poli P, Borgwardt S, Bechdolf A, Addington J, Riecher-Rössler A, Schultze-Lutter F (2013). The psychosis high-risk state: a comprehensive state-of-the-art review. Arch Gen Psychiatry.

[7] Snyder HR (2013). Major depressive disorder is associated with broad impairments on neuropsychological measures of executive function: a meta-analysis and review. Psychol Bull.

[8] Lee RSC, Hermens DF, Porter MA, Redoblado-Hodge MA (2012). A meta-analysis of cognitive deficits in first-episode major depressive disorder. J Affect Disord.

[9] Martin DM, Wollny-Huttarsch D, Nikolin S, McClintock SM, Alonzo A, Lisanby SH (2020). Neurocognitive subgroups in major depressive disorder. Neuropsychology..

[10] Russo M, Van Rheenen TE, Shanahan M, Mahon K, Perez-Rodriguez MM, Cuesta-Diaz A (2017). Neurocognitive subtypes in patients with bipolar disorder and their unaffected siblings. Psychol Med.

[11] Bora E, Hıdıroğlu C, Özerdem A, Kaçar ÖF, Sarısoy G, Civil Arslan F (2016). Executive dysfunction and cognitive subgroups in a large sample of euthymic patients with bipolar disorder. Eur Neuropsychopharmacol.

[12] Burdick KE, Russo M, Frangou S, Mahon K, Braga RJ, Shanahan M (2014). Empirical evidence for discrete neurocognitive subgroups in bipolar disorder: Clinical implications. Psychol Med.

[13] Haas SS, Ge R, Sanford N, Modabbernia A, Reichenberg A, Whalley HC, et al. Accelerated global and local brain aging differentiate cognitively impaired from cognitively spared patients with schizophrenia. Front. Psychiatry. 2022;13:913470. 10.3389/fpsyt.2022.913470 PMC9257006 35815015

[14] Wenzel J, Haas SS, Dwyer DB, Ruef A, Oeztuerk OF, Antonucci LA, et al. Cognitive subtypes in recent onset psychosis: distinct neurobiological fingerprints? Neuropsychopharmacology. 2021. 10.1038/s41386-021-00963-1. 10.1038/s41386-021-00963-1 PMC8209013 33723384

[15] Van Rheenen TE, Cropley V, Zalesky A, Bousman C, Wells R, Bruggemann J (2018). Widespread volumetric reductions in schizophrenia and schizoaffective patients displaying compromised cognitive abilities. Schizophr Bull.

[16] Karantonis JA, Rossell SL, Carruthers SP, Sumner P, Hughes M, Green MJ (2020). Cognitive validation of cross-diagnostic cognitive subgroups on the schizophrenia-bipolar spectrum. J Affect Disord.

[17] Van Rheenen TE, Lewandowski KE, Tan EJ, Ospina LH, Ongur D, Neill E (2017). Characterizing cognitive heterogeneity on the schizophrenia-bipolar disorder spectrum. Psychol Med.

[18] Cotrena C, Damiani Branco L, Ponsoni A, Milman Shansis F, Paz Fonseca R (2017). Neuropsychological clustering in bipolar and major depressive disorder. J Int Neuropsychol Soc.

[19] Iverson GL, Brooks BL, Langenecker SA, Young AH (2011). Identifying a cognitive impairment subgroup in adults with mood disorders. J Affect Disord.

[20] Haining K, Gajwani R, Gross J, Gumley AI, Ince RAA, Lawrie SM, et al. Characterising cognitive heterogeneity in individuals at clinical high-risk for psychosis: a cluster analysis with clinical and functional outcome prediction. Eur Arch Psychiatry Clin Neurosci. 2021. 10.1007/s00406-021-01315-2. 10.1007/s00406-021-01315-2 PMC8938352 34401957

[21] Abramovitch A, Short T, Schweiger A (2021). The C factor: cognitive dysfunction as a transdiagnostic dimension in psychopathology. Clin Psychol Rev.

[22] Lima-Ojeda JM, Rupprecht R, Baghai TC (2018). Neurobiology of depression: a neurodevelopmental approach. World J Biol Psychiatry.

[23] Murray RM, Bhavsar V, Tripoli G, Howes O (2017). 30 years on: how the neurodevelopmental hypothesis of schizophrenia morphed into the developmental risk factor model of psychosis. Schizophr Bull.

[24] Weinberg D, Lenroot R, Jacomb I, Allen K, Bruggemann J, Wells R (2016). Cognitive subtypes of schizophrenia characterized by differential brain volumetric reductions and cognitive decline. JAMA Psychiatry.

[25] Gould IC, Shepherd AM, Laurens KR, Cairns MJ, Carr VJ, Green MJ (2014). Multivariate neuroanatomical classification of cognitive subtypes in schizophrenia: a support vector machine learning approach. NeuroImage Clin.

[26] Woodward ND, Heckers S (2015). Brain structure in neuropsychologically defined subgroups of schizophrenia and psychotic bipolar disorder. Schizophr Bull.

[27] Lewandowski KE, McCarthy JM, Öngür D, Norris LA, Liu GZ, Juelich RJ (2019). Functional connectivity in distinct cognitive subtypes in psychosis. Schizophr Res.

[28] Yang X, Qi S, Wang M, Calhoun VD, Sui J, Li T (2021). Subtypes of depression characterized by different cognitive decline and brain activity alterations. J Psychiatr Res.

[29] Kambeitz J, Kambeitz-Ilankovic L, Leucht S, Wood S, Davatzikos C, Malchow B (2015). Detecting neuroimaging biomarkers for schizophrenia: a meta-analysis of multivariate pattern recognition studies. Neuropsychopharmacology..

[30] Koutsouleris N, Kambeitz-Ilankovic L, Ruhrmann S, Rosen M, Ruef A, Dwyer DB (2018). Prediction models of functional outcomes for individuals in the clinical high-risk state for psychosis or with recent-onset depression: a multimodal, multisite machine learning analysis. JAMA Psychiatry.

[31] Miller TJ, McGlashan TH, Rosen JL, Cadenhead K, Cannon T, Ventura J (2003). Prodromal assessment with the structuredinterview for prodromal syndromesand the scale of prodromal symptoms:predictive validity, interrater reliability,and training to reliability. Schizophr Bull.

[32] Schultze-Lutter, F, Addington, J, Ruhrmann, S, Klosterkötter J. Schizophrenia proneness instrument, adult version (SPI-A). Rome: Rome: Giovanni Fioriti; 2007.

[33] Hall RCW (1995). Global assessment of functioning: a modified scale. Psychosomatics..

[34] Kay SR, Fiszbein A, Opler LA (1987). The positive and negative syndrome scale (PANSS) for schizophrenia. Schizophr Bull.

[35] Beck AT, Ward CH, Mendelson M, Mock J, Erbaugh J. An inventory for measuring depression. Arch Gen Psychiatry. 1961;4:561–71. 10.1001/archpsyc.1961.01710120031004 13688369

[36] Nuechterlein KH, Green MF, Kern RS, Baade LE, Barch DM, Cohen JD (2008). The MATRICS consensus cognitive battery, part 1: test selection, reliability, and validity. Am J Psychiatry.

[37] Kern RS, Nuechterlein KH, Green MF, Baade LE, Fenton WS, Gold JM (2008). The MATRICS consensus cognitive battery, Part 2: Co-norming and standardization. Am J Psychiatry.

[38] Rey A (1941). L’examin psychologique dans les casd’escephalopathie traumatique. Arch Psychol (Geneve).

[39] Osterrieth PA (1944). Le test de copie d’unefigurecomplexe. Arch Psychol (Geneve).

[40] Nowicki S, Duke MP (1994). Individual differences in the nonverbal communication of affect: the diagnostic analysis of nonverbal accuracy scale. J Nonverbal Behav.

[41] Wechsler D. Manual for the Wechsler Adult Intelligence Scale. Oxford, England: Psychological Corp.; 1955.

[42] Petrides M, Milner B (1982). Deficits on subject-ordered tasks after frontal- and temporal-lobe lesions in man. Neuropsychologia..

[43] Ruff RM, Light RH, Parker SB, Levin HS (1996). Benton controlled Oral Word Association Test: reliability and updated norms. Arch Clin Neuropsychol.

[44] Tombaugh TN (2004). Trail making Test A and B: normative data stratified by age and education. Arch Clin Neuropsychol.

[45] Schmidt M. Rey auditory verbal learning test: a handbook (Vol. 17). Los Angeles, CA: Western Psychological Services; 1996.

[46] Eliason MJ, Richman LC (1987). The continuous performance test in learning disabled and nondisabled children. J Learn Disabil.

[47] Kapur S (2003). Psychosis as a state of aberrant salience: a framework linking biology, phenomenology, and pharmacology in schizophrenia. Am J Psychiatry.

[48] Benjamini Y, Hochberg Y (1995). Controlling the false discovery rate: a practical and powerful approach to multiple testing. J R Stat Soc Ser B.

[49] Hennig C (2007). Cluster-wise assessment of cluster stability. Comput Stat Data Anal.

[50] Hennig C. fpc: flexible procedures for clustering (Version 2.2-9). 2020.

[51] Haas SS, Antonucci LA, Wenzel J, Ruef A, Biagianti B, Paolini M (2020). A multivariate neuromonitoring approach to neuroplasticity-based computerized cognitive training in recent onset psychosis. Neuropsychopharmacology..

[52] Dosenbach NUF (2010). Prediction of Individual Brain. Science (80-).

[53] Millan MJ, Agid Y, Brüne M, Bullmore ET, Carter CS, Clayton NS (2012). Cognitive dysfunction in psychiatric disorders: characteristics, causes and the quest for improved therapy. Nat Rev Drug Discov.

[54] Cannon TD, Yu C, Addington J, Bearden CE, Cadenhead KS, Cornblatt BA (2016). An individualized risk calculator for research in prodromal psychosis. Am J Psychiatry.

[55] Zanelli J, Reichenberg A, Sandin S, Morgan C, Dazzan P, Pilecka I (2022). Dynamic and static cognitive deficits in schizophrenia and bipolar disorder after the first episode. Schizophr Bull.

[56] Dickinson D, Zaidman SR, Giangrande EJ, Eisenberg DP, Gregory MD, Berman KF (2020). Distinct polygenic score profiles in schizophrenia subgroups with different trajectories of cognitive development. Am J Psychiatry.

[57] Green MJ, Cairns MJ, Wu J, Dragovic M, Jablensky A, Tooney PA (2013). Genome-wide supported variant MIR137 and severe negative symptoms predict membership of an impaired cognitive subtype of schizophrenia. Mol Psychiatry.

[58] Wells R, Swaminathan V, Sundram S, Weinberg D, Bruggemann J, Jacomb I, et al. The impact of premorbid and current intellect in schizophrenia: cognitive, symptom, and functional outcomes. Npj Schizophr. 2015;1:1–8. 10.1038/npjschz.2015.43 PMC4849463 27336046

[59] Lewandowski KE, Sperry SH, Cohen BM, Öngür D (2014). Cognitive variability in psychotic disorders: a cross-diagnostic cluster analysis. Psychol Med.

[60] Velthorst E, Meyer EC, Giuliano AJ, Addington J, Cadenhead KS, Cannon TD (2019). Neurocognitive profiles in the prodrome to psychosis in NAPLS-1. Schizophr Res.

[61] Carruthers SP, Van Rheenen TE, Karantonis JA, Rossell SL. Characterising demographic, clinical and functional features of cognitive subgroups in schizophrenia spectrum disorders: a systematic review. Neuropsychol Rev. 2021. 10.1007/s11065-021-09525-0. 10.1007/s11065-021-09525-0 34694542

[62] Cambridge OR, Knight MJ, Mills N, Baune BT (2018). The clinical relationship between cognitive impairment and psychosocial functioning in major depressive disorder: a systematic review. Psychiatry Res.

[63] Keilp JG, Madden SP, Gorlyn M, Burke AK, Oquendo MA, Mann JJ (2018). The lack of meaningful association between depression severity measures and neurocognitive performance. J Affect Disord.

[64] McClintock SM, Husain MM, Greer TL, Cullum CM (2010). Association between depression severity and neurocognitive function in major depressive disorder: a review and synthesis. Neuropsychology..

[65] Zaninotto L, Solmi M, Veronese N, Guglielmo R, Ioime L, Camardese G (2016). A meta-analysis of cognitive performance in melancholic versus non-melancholic unipolar depression. J Affect Disord.

[66] Chung BS, Cannon TD (2015). Brain imaging during the transiation from psyhcosis prodrome t schizpohrenia. Physiol Behav.

[67] Zhang FF, Peng W, Sweeney JA, Jia ZY, Gong QY (2018). Brain structure alterations in depression: psychoradiological evidence. CNS Neurosci Ther.

[68] Wang L, Hermens DF, Hickie IB, Lagopoulos J (2012). A systematic review of resting-state functional-MRI studies in major depression. J Affect Disord.

[69] Durstewitz D, Koppe G, Meyer-Lindenberg A (2019). Deep neural networks in psychiatry. Mol Psychiatry.

[70] McTeague LM, Huemer J, Carreon DM, Jiang Y, Eickhoff SB, Etkin A (2017). Identification of common neural circuit disruptions in cognitive control across psychiatric disorders. Am J Psychiatry.

