# Supplementary material for: Transdiagnostic subgroups of cognitive impairment in early affective and psychotic illness
Source: Neuropsychopharmacology. 2023 Sep 22;49(3):573–83. doi: 10.1038/s41386-023-01729-7 (PMC10789737; doi:10.1038/s41386-023-01729-7)
Supplement: Supplementary file 1 — Supplementary Material [file 41386_2023_1729_MOESM1_ESM.docx]

**Supplementary Material**

[Methods 3](#_Toc141974696)

[Inclusion and exclusion criteria 3](#_Toc141974697)

[Quality control and exclusion of study participants 4](#_Toc141974698)

[Quality control and exclusion of cognitive variables 4](#_Toc141974699)

[Clustering and cognitive variable preprocessing 4](#_Toc141974700)

[Calculation of salience score for cognitive variables 4](#_Toc141974701)

[Harmonization of verbal learning scores 5](#_Toc141974702)

[Dimensionality reduction for cognitive variables using principal component analysis (PCA) 5](#_Toc141974703)

[Clustering algorithm, cluster number and cluster stability assessment 6](#_Toc141974704)

[Replication sample preprocessing and external cluster projection 6](#_Toc141974705)

[Neuroimaging and supervised machine learning pipeline 7](#_Toc141974706)

[Structural MRI (sMRI) preprocessing pipeline 7](#_Toc141974707)

[Resting-state functional MRI (rsfMRI) preprocessing pipeline 8](#_Toc141974708)

[Machine learning pipeline: Nested cross-validation framework 8](#_Toc141974709)

[Machine learning pipeline: Feature preprocessing 9](#_Toc141974710)

[Machine learning pipeline: Estimation of voxel reliability across study sites using a G coefficient map 9](#_Toc141974711)

[Machine learning pipeline: Support vector machine (SVM) algorithm and model significance 10](#_Toc141974712)

[Machine learning pipeline: Visualization of discriminative GM volume (GMV) pattern and functional connectivity patterns 10](#_Toc141974713)

[Results 11](#_Toc141974714)

[Discovery sample: Additional cognitive and clinical results 11](#_Toc141974715)

[Discovery sample: GMV results (pattern description) 11](#_Toc141974716)

[Discovery sample: rsFC results (pattern description) 11](#_Toc141974717)

[Post-hoc analysis to characterize relationships between imaging models and behavioral measures 12](#_Toc141974718)

[Replication sample: Additional cognitive and clinical results 12](#_Toc141974719)

[Differences in cognitive performance between transdiagnostic clusters 13](#_Toc141974720)

[Differences in clinical characteristics and functioning levels between transdiagnostic clusters 13](#_Toc141974721)

[Differences in cognitive performance between single-disease clusterings 14](#_Toc141974722)

[Literature 15](#_Toc141974723)

[Tables 17](#_Toc141974724)

[Figures 36](#_Toc141974725)

# Methods

## Inclusion and exclusion criteria

Sites of the discovery sample included the Ludwig Maximilian University of Munich (LMU; N=201), University of Milan (MIL; N=42), University of Basel (BAS; N=98), University of Cologne (UKK; N=141), University of Birmingham (BHAM; N=84), University of Turku (TUR; N= 93) and University of Udine (UD; N=92).

General inclusion criteria were age between 15 and 40 years and sufficient capacity to provide informed consent and follow the neuropsychological instructions and clinical interview. General exclusion criteria comprised (1) history of neurological disease, head trauma with loss of consciousness (> 5minutes), alcoholism or polytoxicomania; (2) intellectual disability based on Wechsler Intelligence Scale for Adults [1]; (3) more than 90 days of antipsychotic treatment in the last year (DGPPN guidelines); (4) any condition violating the magnetic resonance imaging (MRI) safety requirements.

For each diagnostic group specific inclusion criteria were applied [2]. ROP patients were included in the study if they fulfilled DSM-IV-TR criteria for a psychotic episode, present in the last three months, lasting longer than one week and with a first onset in the last 24 months. Recent onset depression (ROD) patients were included if they fulfilled DSM-IV-TR criteria for a first manifestation of a depressive episode, present in the last 3 month and with onset in the last 24 months. CHR status for psychosis was defined as fulfilling attenuated psychotic symptoms (APS) or brief limited intermittent psychotic symptoms (BLIPS) measured by the Structured Interview for Prodromal Syndromes (SIPS) [3], cognitive disturbances (COGDIS) measured by the Schizophrenia Proneness Instrument (SPIA) [4] or positive family history (1st degree relatives) for psychosis and a more than 30% drop in functioning in the last 6 month compared to highest functioning for life time. HC volunteers had not to fulfill any current or past DSM-IV-TR axis I or II diagnosis and/or clinical high-risk status for psychosis. Study groups fulfilled specific criteria with respect to intake of antipsychotic medication in accordance with the DGPPN S3 Guidelines for treatment of first-episode psychosis.

Further, we followed specific criteria with respect to intake of antipsychotic medication. ROP patients with intake of antipsychotic medication of more than 90 cumulative days (dosage >= minimum dosage threshold of DGPPN S3 Guidelines for the treatment of first-episode psychosis) and/or any intake of antipsychotic medication in the same dosage within the past 3 months before study enrollment were excluded. ROD patients and HC volunteers with intake of antipsychotic medication of more than 30 cumulative days (dosage higher than a minimum dosage threshold of DGPPN S3 Guidelines for the treatment of first-episode psychosis) and/or any intake of antipsychotic medication in the same dosage within the past 3 months before study enrollment were excluded. HC with intake of psychotropic medications or drugs exceeding 5 occasions a year were excluded.

## Quality control and exclusion of study participants

Study participants in the discovery sample were excluded due to the following reasons: not fulfilling inclusion criteria, no imaging data acquired, showing more than 50% of missing values across all cognitive variables. Further, we inspected outliers for each study group separately and excluded all participants showing values more than 3 SD above or below the variable mean in more than 15% of the cognitive variables (Figure S1). We followed the same procedure in the replication sample.

## Quality control and exclusion of cognitive variables

To reduce the dimensionality and to avoid biases due to a high number of missing values of the cognitive data (which need to be imputed as a requirement for the clustering analysis) we proceeded as follows (Figure S1). First, we excluded each variable that contained ‘task-inherent’ missings, i.e. a variable that only holds a value, if a participant reacted in certain way, but otherwise is missing. For example, the variable ‘CPTIP_Reaction_times_error_distracting_50_trials_T0’ represents the reaction time of a participant when a distracting stimulus was displayed in the continuous performance task (CPTIP). It only holds a value, if the participant made an error on a distracting stimulus. Second, we excluded two variables with > 60% missing values. Finally, we excluded variables showing low variance. We matched the cognitive variables of the replication sample with the discovery sample. Missing values of the cognitive data prior to imputation and further statistical processing are displayed in Figure S2.

## Clustering and cognitive variable preprocessing

### Calculation of salience score for cognitive variables

We generated measures of implicit adaptive and aberrant salience and explicit adaptive and aberrant salience according to previous studies [5]. Explicit adaptive salience was calculated as the mean of the participant’s probability estimate of blue animals and blue objects subtracted from the mean of the participant’s probability estimate of red animals and red objects. Explicit aberrant salience was calculated as the absolute value of the participant’s mean probability estimate of blue animals and blue objects subtracted from the participant’s mean probability estimate of blue objects and red objects. In a first step standardized reaction times for blue and red items and animals and objects were calculated (e.g. reaction time for blue items subtracted from the total mean reaction time divided by the total reaction time standard deviation) for the implicit salience measures. Implicit adaptive salience was calculated by subtracting the standardized reaction times of blue items from the standardized reaction times of red items and, analogously, for implicit aberrant salience the same procedure was applied with respect to animals and objects.

For further analysis we kept only the calculated scores.

### Harmonization of verbal learning scores

PRONIA study site Turku used the Hopkins Verbal Learning Task – Revised (HVLT) [6] whereas all other sites used Rey Auditory Verbal Learning Task (RAVLT) [7] for assessment of verbal memory performance. As outlined in previous work of the PRONIA consortium [8], the PRONIA consortium generated a regression model which can be used to translate the sum score of 3 trials in the HVLT to the sum score of 5 trials contained within the RAVLT: RAVLT_5sum_= 25.51264904*HAVLT_3sum_ + 1.191092045. This formula was used in the current sample to include the verbal memory performance of all study sites in the analysis.

We included four variables in the further analyses: The harmonized sum of correctly named words across all word lists, the total repetition of words across all word lists, the sum of correctly named words of the first word list and the sum of correctly named words for delayed recall.

### Dimensionality reduction for cognitive variables using principal component analysis (PCA)

The statistical analysis comprised the following steps. First, we imputed the remaining missing values by variable median separately for each study group. Second, we regressed out effects of age, sex, years of education and study site in the patient sample by using multiple linear regression models generated on the HC data set. Third, we reduced the dimensionality of the analyzed cognitive features by conducting separate PCAs on groups of scaled cognitive variables assigned to a certain cognitive domain (Table S2) and retaining the each first principal component (PC) for further analyses. This procedure was applied only to patients/CHR individuals and resulted in one variable (first PC) for each cognitive domain (visual memory, social cognition, working memory, processing speed, verbal memory, executive functioning, attention and salience) capturing the highest proportion of variance (Figure S3).

We projected the obtained cognitive domains of the discovery sample into the data space of the preprocessed HC of the discovery sample by multiplication of the HC data matrices with the PCA weights of the discovery sample to generate comparable cognitive domains in the HC dataset. Similarly, we projected the discovery sample cognitive domains into the preprocessed cognitive variables of the replication sample to investigate the generalization of the clustering model. We followed the same approach for the individual clusterings.

We associated each cognitive domain variable with their underlying original neuropsychological variables through correlation to interpret their directionality through majority vote (high values of the cognitive domain variable relate to high performance or v.v.). Cognitive domain variables were anti-poled for visualization to consistently represent higher values with higher performance after the clustering procedure. We scaled the eight remaining principal components (referring to the eight cognitive domains in the data set) before clustering.

### Clustering algorithm, cluster number and cluster stability assessment

We applied K-means clustering on the eight cognitive domain variables generated by the PCA. K-means clustering is an unsupervised machine learning algorithm which partitions a given data set into k predefined subgroups by minimizing the variation within a given subgroup, i.e., the distance of individual observations to the cluster centroid [9]. To decide on the optimal number of k clusters, we ran the algorithm with random initializations (N=1000) over a cluster range of 2 to 10 and used the Calinski-Harabasz index [10] and the average silhouette width [11] to evaluate their between-cluster separation and within-cluster closeness. Subsequently, clustering was embedded in a resampling procedure to determine cluster stability on the selected cluster number using two independent strategies (‘subset’ and ‘noise’) based on the Jaccard index (56)[12] (Figure S4).

## Replication sample preprocessing and external cluster projection

For the replication sample, we retained the identical set of cognitive variables as in the discovery sample. We followed the same quality control, exclusion procedures and preprocessing as in the discovery sample.

We regressed out effects of sex, age, years in education and site based on a linear model generated on the HC sample. To obtain comparable cognitive domain scores, we projected the discovery sample cognitive domains into the data space of the preprocessed cognitive variables of the replication sample. Finally, we predicted the cluster assignment of each observation in the replication sample based on the discovery cluster model. We followed the same approach for the individual study group subsets in the replication sample.

## Neuroimaging and supervised machine learning pipeline

### Structural MRI (sMRI) preprocessing pipeline

Grey matter (GM) structural brain images were inspected for scanner artifacts and anatomical abnormalities by trained personnel and radiologists at each study site independently. To retain clinical real-world scanner diversity, PRONIA uses a minimal harmonization of images procedure allowing variation in scan parameters across scanning sites under the criteria of optimal signal-to-noise ratio and contrast between cortical ribbon and white matter. Scanner parameters are detailed in Koutsouleris et al. (2018).

Preprocessing followed the protocols established in Koutsouleris et al. (2018) and the CAT12 manual (www.neuro.uni-jena.de/cat12/CAT12-Manual.pdf). Imaging data was preprocessed using the open-source CAT12 toolbox (version >r1200; http://dbm.neuro.uni-jena.de/cat12/), an extension of the SPM12 software (Wellcome Department of Cognitive Neurology, London, UK;<http://www.fil.ion.ucl.ac.uk/spm/software/spm12/>). In accordance with previous studies [2] and the CAT12 manual ([www.neuro.uni-jena.de/cat12/CAT12-Manual.pdf](http://www.neuro.uni-jena.de/cat12/CAT12-Manual.pdf)) preprocessing was based on the following steps: (1) denoising based on Spatially Adaptive Non-Local Means (SANLM) filtering [13]; (2) adjusting the images for white matter inhomogeneities and varying GM intensities using Local Adaptive Segmentation (LAS) [14]; (3) segmentation across cortical and subcortical structures by means of an Adaptive Maximum A Posteriori (AMAP) segmentation technique; (4) additional denoising of the AMAP segmentation estimation using a Markov Random Field approach [14]; (5) application of a partial volume segmentation algorithm to model different tissue intensities (GM, white matter, cerebrospinal fluid) in the AMAP-generated tissue segments; (6) coregistration of the images to a MNI-template generated from the MRI data of 555 healthy controls in the IXI database (<http://www.braindevelopment.org>). Images were quality controlled using the CAT12 toolbox by correlating brain images among each other to check for signal homogeneity and by visual inspection. No cases had to be excluded.

### Resting-state functional MRI (rsfMRI) preprocessing pipeline

The resting-state functional MRI (rsfMRI) pipeline comprised two general steps following the protocol established in Haas et al. (2020): core and denoising steps [15].

Core steps were conducted using Statistical Parametric Mapping, version 12 (SPM12) (<https://www.fil.ion.ucl.ac.uk/spm/software/spm12/>; version 6685). Images were slice time corrected, unwarped and realigned to the first volume for head-motion correction. Furthermore, we calculated framewise displacement (FD) parameters [16] to evaluate the amount of movement of each participant. We excluded all participants (N=13) with more than 38.5% of volumes being displaced for more than 0.5 mm [16]. To further correct images for head motion, we used the FD parameters as covariate in the ML analysis. After that, images were co-registered and normalized to MNI space. Finally, functional volumes were masked using the GM mask and images were smoothed using a Gaussian kernel of 6 mm full width at half-maximum.

Denoising steps included background and temporal band-pass filtering (0.01 – 0.08 Hz) and confound signal regression based on the Friston 24 motion parameters serving as motion estimates. Furthermore, participant motion during the scan was corrected using time series despiking (Wavelet Despike) with the BrainWavelet Toolbox ([www.brainwavelet.org/)](http://www.brainwavelet.org/)%5b52) [15].

The preprocessed voxel-space rsfMRI data was parcellated into 160 regions of interest (ROIs) according to the Dosenbach functional atlas [17] and the mean signal was extracted from 10mm spheres centered at each ROI using the MarsBaR Toolbox version 0.42 [18]. We calculated pairwise Pearson’s correlations of the average time series between ROIs using in-house scripts running in Matlab R2015. The procedure resulted in connectivity matrices of 12720 resting-state functional connectivity (rsFC) features per participant.

### Machine learning pipeline: Nested cross-validation framework

Similarly as in previous work [2,19,20] we embedded our classification analysis into a nested cross-validation scheme to (1) minimize the probability to overfit the model, (2) evaluate within sample generalizability to data unseen from the classification model and (3) optimise model parameters to increase classification performance. This was done separately for structural brain images and resting state connectivity matrices.

The following steps of the cross-validation procedure were repeated for all folds and permutation of the data set:

1) SVM models were calculated on the inner (CV1) training sample and evaluated on the held out inner test sample separately for each ‘fold-permutation-parameter’-combination.

2) Majority vote selected the optimal parameter combination based on the model’s balanced accuracies (BAC).

3) The whole inner loop data (training, test) was retrained on the selected parameter combination.

4) The final model was validated and assessed based on the held-out outer (CV2) test sample.

### Machine learning pipeline: Feature preprocessing

Prior to classification GM images were preprocessed and optimized. Steps included (1) 6mm gaussian kernel smoothing, (2) correction for total intracranial volume (TIV), (3) removal of zero-variance voxels, (4) masking of unreliable voxels across scanning sites using a G coefficient map (section below) [2], (5) reducing image dimensionality by PCA (80% of component retention) and (6) scaling.

Prior to classification functional connectivity matrices were (1) pruned for zero-variance and completely missing features, (2) corrected for framewise displacement using a partial correlations approach, (3) pruned by applying PCA retaining 25%, 50% and 75% of the components and (4) scaled.

### Machine learning pipeline: Estimation of voxel reliability across study sites using a G coefficient map

A G coefficient map was calculated to estimate the reliability of grey matter (GM) voxels across the PRONIA study sites using the data of HC traveling subjects (N=6), i.e., subjects scanned at each MRI acquisition site [2]. Using the G coefficient map GM voxels highly variable in their signal across site were masked out prior to the application of the classification algorithm to control for site-specific scanner noise and differences in MRI protocols.

### Machine learning pipeline: Support vector machine (SVM) algorithm and model significance

For all SVM analyses we fit a hyperplane by applying a linear support vector machine (SVM) algorithm optimizing on 11 different c-parameter settings (range: 2^-3^ to 2^4^) to strike a balance between penalization for misclassification and generalizability. In addition, model parameters were weighted for uneven group sizes. Classification performance was evaluated based on balanced accuracy (BAC).

As implemented in the NM toolbox we used permutation (N_perm_= 100; alpha = 0.05) for significance testing of the final model resulting from the cross-validation procedure by permuting the labels [2,21]. We calculated ANOVAs to assess the effect of clinical and demographic variables on the model’s decision scores.

### Machine learning pipeline: Visualization of discriminative GM volume (GMV) pattern and functional connectivity patterns

We evaluated the reliability of voxels contributing to the classification performance of the winning model by visualizing the cross-validation ratio (CV ratio). The CV ratio represents the mean CV2 weight of all CV2 folds and permutations divided by the CV1 standard error for a given voxel (GMV analysis)/correlation between two brain regions (rsFC analysis) of the winning model. Higher/lower weights indicate a stronger contribution to the classification performance for one group or the other. The directionality of the weight (positive or negative) indicates whether a given voxel is predictive of one group or the other [2].

# Results

Both transdiagnostic and individual study group clusterings indicated a two clustering solution as the optimal fit for the data (Figure S4). Jaccard similarity indices [22] revealed a high stability of the obtained clusters for both applied resampling methods across the transdiagnostic and individual subgroup clusterings (Figure S4). For the transdiagnostic clustering we observed Jaccard values of 0.85 (cluster 1) and 0.91 (cluster 2) for the subset method and 0.89 (cluster 1) and 0.94 (cluster 2) for the noise method. For the ROP clustering we observed Jaccard values of 0.82 (cluster 1) and 0.91 (cluster 2) for the subset method and 0.90 (cluster 1) and 0.95 (cluster 2) for the noise method. For the ROD clustering we observed Jaccard values of 0.87 (cluster 1) and 0.78 (cluster 2) for the subset method and 0.90 (cluster 1) and 0.95 (cluster 2) for the noise method. For the CHR clustering we observed Jaccard values of 0.86 (cluster 1) and 0.88 (cluster 2) for the subset method and 0.90 (cluster 1) and 0.91 (cluster 2) for the noise method.

## Discovery sample: Additional cognitive and clinical results

## Discovery sample: GMV results (pattern description)

In the ‘spared vs HC’-model, voxels predictive of spared cluster status (positive CV ratio) were located in the pedunculi area of right cerebellum, in left calcarine fissure/cingulum, left postcentral gyrus, left putamen and left fusiform gyrus. Predictive voxels for HC status (negative CV ratio) mainly were observed in the left anterior cingulum. Further, smaller groups of predictive voxels were located in the left and right inferior temporal cortex, right parahippocampal gyrus and rolandic operculum.

## Discovery sample: rsFC results (pattern description)

Both significant classification models showed restricted and mainly posterior connectivity patterns when predicting impaired or spared cluster status. In contrast, the prediction of HC status often involved long-range, inter-hemispheric connections from parietal and occipital to frontal areas. Particularly prominent were several subcortical thalamic-cerebellar connections predictive of HC status in the ‘impaired vs HC’ model. Further, we identified cortical-subcortical connectivity patterns. Connectivity between thalamus and temporal areas, basal ganglia and parietal areas were predictive of impaired cluster status and connectivity between thalamus and prefrontal cortex were predictive of spared cluster status.

## Post-hoc analysis to characterize relationships between imaging models and behavioral measures

In post-hoc analyses we evaluated the effects of demographic, clinical and functional variables on the absolute decision scores (indicating the reliability of classification) for the significant structural and functional connectivity classification models by calculating correlational and Analyses of Variance (ANOVA).

Decision scores of the GMV model classifying spared subgroup and HC were non-significantly correlated to age (t(507) = -0.867, p = 0.3861, r = -0.038) and unrelated to sex (t(472,21) = 0.997, p = 0.319), study site (F(6,502) = 1.079, p = 0.374) and study group (F(3, 505) = 1.273, p = 0.283). However, decision scores showed a small but significant correlation for GAF in the month prior to study entry (t(504) = - 1.984, p < 0.05, r = - 0.088), in the year before study entry (t(504) = -2.999, p < 0.01, r = - 0.132) and across life time (t(594) = -2.590, p < 0.01, r = - 0.114) indicating a higher reliability in classification for individuals with lower GAF scores.

Decision scores of the rsFC model classifying impaired subgroup and HC were non-significantly correlated to age (t(403) = 0.931, p = 0.352, r = 0.046), to any GAF measure (month prior to study entry: t(399) = 0.002, p = 0.998, r = 0.0001; year prior to study entry: t(399) = - 0.561, p = 0.575, r = -0.028; across life time: t(399) = -0.277, p = 0.782, r = -0.014). Further, decision scores were unrelated to sex (t(401.98) = -1.071, p = 0.285), study site (F(6,398) = 1.380, p = 0.221), study group (F(3,401) = 0.513, p = 0.674).

Decision scores of the rsFC model classifying spared subgroup and HC were non-significantly correlated to age (t(507) = 0.356, p = 0.722, r = 0.016), GAF in the year before study entry (t(504) = -1.207, p = 0.228, r = -0.054) and across life time (t(504) = -1.105, p = 0.270, r = -0.049) and unrelated to sex (t(488.48) = -0.562, p = 0.574) and study group (F(3,505) = 1.954, p = 0.12). However, we found a small but significant correlation with GAF in the month prior to study entry (t(504) = -2.002, p < 0.05, r = -0.089) indicating a higher reliability in classification for individuals with lower GAF scores and a significant association with study site (F(6,502) = 7.18, p < 0.001) indicating particularly low decision scores for site Turku and particularly high reliability for site Milan.

## Replication sample: Additional cognitive and clinical results

The projection of the transdiagnostic discovery sample clusters on the replication sample led to 161 individuals being assigned to the impaired cluster (ROP = 81 (50%), ROD = 32 (20%), CHR = 48 (30%)) and 128 individuals being assigned to the spared cluster (ROP = 27 (21%), ROD = 49 (38%), CHR = 52 (41%)). All study groups were represented in both clusters though ROP patients were more frequent in the impaired subgroup and ROD patients in the spared subgroup (X^2^(1,283) = 27.32, p < 0.001) (Table S13).

### Differences in cognitive performance between transdiagnostic clusters

Similar to the findings in the discovery sample, the impaired cluster indicated significantly worse performance in comparison to the spared cluster across all cognitive domains including salience (F(2,406) > 6.99, p < 0.001: for all domains at least: p < 0.01). Whereas the impaired cluster performed significantly worse than HC across all cognitive domains (for all domains at least: p < 0.01), the spared cluster performed comparable to HC with respect to processing speed (p = 0.30), executive functioning (p = 0.31), attention (p = 0.087) and visual memory (p = 0.58) and better than HC in social cognition (p < 0.01), working memory (p < 0.01), and verbal memory (p < 0.05). The spared cluster performed worse than HC with respect to salience (p < 0.01)(Table 2; Figure S6).

The impaired cluster showed a significantly lower vocabulary (main effect: cluster 1/cluster 2/HC: F(2,406) = 25.241, p < 0.001) and matrix score (main effect: cluster 1/cluster 2/HC: F(2,406) = 18.632, p < 0.001) in comparison to the spared cluster (vocabulary: p < 0.001; matrix: p < 0.001) and HC (vocabulary: p < 0.001; matrix: p < 0.001). In contrast, the spared cluster showed significantly lower vocabulary score (p < 0.01) but comparable matrix score (p = 0.28) relative to HC.

### Differences in clinical characteristics and functioning levels between transdiagnostic clusters

Impaired and spared cluster showed significantly lower functioning with respect to the GAF symptom scale in the year (main effect: cluster 1/cluster 2/HC: F(2, 399) = 185.02, p < 0.001) and month (main effect: cluster 1/cluster 2/HC: F(2, 399) = 486.93, p < 0.001) prior to study entry as well as across the life span (main effect: cluster 1/cluster 2/HC: F(2, 399) = 58.323, p < 0.001) when compared to HC (impaired: p < 0.001; spared: p < 0.001). However, the impaired cluster showed significantly lower functioning with respect to the GAF symptom scale only in the month prior to study entry (p < 0.01). Functioning results with respect to the GAF disability scale follow the same pattern (Table S13, Figure S7).

The impaired cluster in comparison to the spared cluster showed significantly higher positive (t(270.63) = 4.603, p < 0.001) symptoms on the PANSS scale. Both impaired and spared cluster showed significantly lower depressive symptoms on the BDI as compared to HC (main effect: cluster 1/cluster 2/HC: F(2, 346) = 128.659, p < 0.001; impaired vs HC: p < 0.001; spared vs HC: p < 0.001) while the spared subgroup showed higher depressive symptoms in comparison to the impaired subgroup (p < 0.05).

### Differences in cognitive performance between single-disease clusterings

Projecting the single-disease clustering solutions on the replication sample we identified 56 (52%) impaired individuals and 52 (48%) spared individuals in the ROP clustering, 42 (52%) impaired individuals and 39 (48%) spared individuals in the ROD clustering, and 63 (63%) impaired and 37 (37%) spared individuals in the CHR clustering (Table S14-S16). Similar to the transdiagnostic cluster solution of discovery and replication sample, the individual clusterings showed an impaired subgroup with widespread reductions in cognitive performance relative to HC and a spared subgroup often performing similar to or better than HC.

When comparing impaired and spared subgroups across clusterings, we found that the impaired and spared subgroup of the ROP clustering performed significantly worse in comparison to the impaired and spared subgroups of ROD and CHR in the domains of working memory (main effect study group: F(2, 283) = 5.825, p < 0.01; impaired ROD: p < 0.01; impaired CHR: p < 0.05), processing speed (main effect study group: F(2, 283) = 11.230, p < 0.001; impaired ROD: p < 0.001; impaired CHR: p < 0.001), attention (main effect study group: F(2, 283) = 12.503, p < 0.001; impaired ROD: p < 0.001; impaired CHR: p < 0.01), executive functioning (main effect study group: F(2, 283) = 5.392, p < 0.01; impaired ROD: p < 0.01) and verbal memory (main effect study group: F(2, 283) = 9.449, p < 0.001; impaired ROD: p < 0.01; impaired CHR: p < 0.01). Additionally, we obtained a significant interaction for visual memory (interaction effect: F(2, 283) = 7.758, p < 0.001) showing that the impaired ROP group performed significantly worse as compared to impaired ROD (p < 0.001) and CHR (p < 0.001) whereas the spared ROP group performed comparable to the spared ROD (p = 0.672) and CHR group (p = 0.856) (Table S7).

# Literature

1. Wechsler D. Manual for the Wechsler Adult Intelligence Scale. Oxford, England: Psychological Corp.; 1955.

2. Koutsouleris N, Kambeitz-Ilankovic L, Ruhrmann S, Rosen M, Ruef A, Dwyer DB, et al. Prediction Models of Functional Outcomes for Individuals in the Clinical High-Risk State for Psychosis or with Recent-Onset Depression: A Multimodal, Multisite Machine Learning Analysis. JAMA Psychiatry. 2018;75:1156–1172.

3. Miller TJ, McGlashan TH, Rosen JL, Cadenhead K, Cannon T, Ventura J, et al. Prodromal Assessment With the StructuredInterview for Prodromal Syndromesand the Scale of Prodromal Symptoms:Predictive Validity, Interrater Reliability,and Training to Reliability. Schizophr Bull. 2003;29:703–716.

4. Schultze-Lutter, F., Addington, J., Ruhrmann, S., Klosterkötter J. Schizophrenia proneness instrument, adult version (SPI-A). Rome: Rome: Giovanni Fioriti; 2007.

5. Roiser JP, Howes OD, Chaddock CA, Joyce EM, McGuire P. Neural and behavioral correlates of aberrant salience in individuals at risk for psychosis. Schizophr Bull. 2013;39:1328–1336.

6. Benedict RHB, Schretlen D, Groninger L, Brandt J. Hopkins verbal learning test - Revised: Normative data and analysis of inter-form and test-retest reliability. Clin Neuropsychol. 1998;12:43–55.

7. Schmidt M. Rey auditory verbal learning test: A handbook (Vol. 17). Los Angeles, CA: Western Psychological Services; 1996.

8. Sanfelici R, Ruef A, Antonucci LA, Penzel N, Sotiras A, Dong M Sen, et al. Novel Gyrification Networks Reveal Links with Psychiatric Risk Factors in Early Illness. Cereb Cortex. 2021:1–12.

9. James G, Witten D, Hastie T, Tibshirani R. An Introduction to Statistical Learning. 2nd ed. New York: Springer US; 2021.

10. Caliñski T, Harabasz J. A Dendrite Method Foe Cluster Analysis. Commun Stat. 1974;3:1–27.

11. Rousseeuw PJ. Silhouettes: A graphical aid to the interpretation and validation of cluster analysis. J Comput Appl Math. 1987;20:53–65.

12. Hennig C. Cluster-wise assessment of cluster stability. Comput Stat Data Anal. 2007;52:258–271.

13. Manjón J V., Carbonell-Caballero J, Lull JJ, García-Martí G, Martí-Bonmatí L, Robles M. MRI denoising using Non-Local Means. Med Image Anal. 2008;12:514–523.

14. Rajapakse JC, Giedd JN, Rapoport JL. Statistical approach to segmentation of single-channel cerebral mr images. IEEE Trans Med Imaging. 1997;16:176–186.

15. Patel AX, Kundu P, Rubinov M, Jones PS, Vertes PE, Ersche KD, et al. A wavelet method for modeling and despiking motion artifacts from resting-state fMRI time series. Neuroimage. 2014;95:287–304.

16. Power JD, Mitra A, Laumann TO, Snyder AZ, Schlaggar BL, Petersen SE. Methods to detect, characterize, and remove motion artifact in resting state fMRI. Neuroimage. 2014;84:320–341.

17. Dosenbach NUF. Prediction of Individual Brain. Science (80- ). 2010;329:1358–1361.

18. Brett M, Anton JL, Valabregue R, Poline JB. Region of interest analysis using the MarsBar toolbox for SPM 99. Neuroimage. 2002;16:294.

19. Wenzel J, Haas SS, Dwyer DB, Ruef A, Oeztuerk OF, Antonucci LA, et al. Cognitive subtypes in recent onset psychosis: distinct neurobiological fingerprints? Neuropsychopharmacology. 2021. 2021. https://doi.org/10.1038/s41386-021-00963-1.

20. Haas SS, Antonucci LA, Wenzel J, Ruef A, Biagianti B, Paolini M, et al. A multivariate neuromonitoring approach to neuroplasticity-based computerized cognitive training in recent onset psychosis. Neuropsychopharmacology. 2020;0:1–8.

21. Golland P, Fischl B. Permutation tests for classification: Towards statistical significance in image-based studies. Lect Notes Comput Sci (Including Subser Lect Notes Artif Intell Lect Notes Bioinformatics). 2003;2732:330–341.

22. Jaccard P. Nouvelles Recherches Sur La Distribution Florale. Bull La Société Vaudoise Des Sci Nat. 1908;44:223–270.

# Tables

**Table S1.** Cognitive variables included in the cluster analysis.

**Table S2.** Neurocognitive tasks and number of included variables for clustering.

| **test name** | **cognitive domain** | **administration** | **Number of variables** |
| --- | --- | --- | --- |
| **Rey-Osterrieth Complex Figure Task (ROCF)** | visual memory | paper-and-pencil format with tablet support | 9 |
| **Diagnostic Analysis of Non-Verbal Accuracy (DANVA-2-AF)** | social cognition | tablet-based | 1 |
| **Auditory Digit Span Task (ADS)** |  |  |  |
| forward trials (-F) | working memory | auditory presentation of numbers by recorded (male) voice | 2 |
| backward trials (-B) | working memory | auditory presentation of numbers by recorded (male) voice | 2 |
| **Verbal Fluency Task (VF)** |  |  |  |
| phonemic trials (-P) | processing speed | named words were recorded and written down by examiner | 5 |
| semantic trials (-S) | processing speed | named words were recorded and written down by examiner | 5 |
| **Hopkins Auditory Verbal Learning Test (HAVLT)** | verbal learning and memory | auditory presentation of word list by recorded (male) voice | 4 |
| **Trail Making Test (TMT)** |  |  |  |
| Trials A (-A) | processing speed | paper-and-pencil format | 1 |
| Trials B (-B) | executive functioning | paper-and-pencil format | 1 |
| **Continuous Perfomance Test, Identical Pairs version (CPT-IP)** | attention and vigilance | tablet-based | 35 |
| **Self-Ordered Pointing Task (SOPT)** | working memory | tablet-based | 13 |
| **Digit Symbol Substitution Test (DSST )** | processing speed | paper-and-pencil format | 2 |
| **Salience Attribution Test (SAT-SV)** | salience | tablet-based | 4 |
| **Wechsler Adult Intelligence Scale (WAIS-III)** |  |  |  |
| Vocabulary | premorbid verbal intelligence | paper-pencil format | 1 |
| Matrices | visual processing and abstract reasoning | paper-pencil format | 1 |

**Table S3.** Demographic and clinical characteristics of the transdiagnostic cluster solution (discovery sample).

|  | **transdiagnostic** | | | | | |
| --- | --- | --- | --- | --- | --- | --- |
|  | **impaired (N=146)** | **spared (N=252)** | **HC (N=270)** | **ANOVA** | | |
|  |  |  |  | **F/t** | **p fdr** | **individual comparisons** |
| **age** | 24.8 (5.4) | 25.38 (5.79) | 25.4 (6.33) | 0.57 | 0.602 | - |
| **sex, male/female** | 81/65 | 129/123 | 105/165 | 13.11 | 0.002 | HC: female > male; imp: male > female |
| **site^1^** | 32/24/26/9/23/14/18 | 96/28/40/24/26/28/10 | 58/39/59/43/23/35/13 | 46.66 | < 0.001 | Milan: imp > sp; Basel/Udine: imp ~ sp; others: sp > imp |
| **studygroup^2^** | 79/30/37 | 61/100/91 | - | 37.20 | < 0.001 | imp: ROP > ROD/CHR; sp: ROD > CHR > ROP |
| **years in education** | 13.96 (3.17) | 14.31 (3.02) | 15.68 (3.22) | 19.00 | < 0.001 | HC > imp; HC > sp |
| **illness duration, days^3^** | 208.66 (172.63) | 202.97 (198.48) | - | 0.25 | 0.803 | - |
| **CPZ^4^** | 461.96 (1170.18) | 218.34 (386.52) | - | 1.48 | 0.162 | - |
| **GAF (symptoms)** |  |  |  |  |  |  |
| lifetime | 77.99 (10.26) | 81.53 (7.72) | 88.04 (5.69) | 92.85 | < 0.001 | imp < sp; HC > sp; HC > imp |
| past year | 62.81 (15.68) | 68.35 (13.67) | 86.97 (6.26) | 253.12 | < 0.001 | imp < sp; HC > sp; HC > imp |
| past month | 46.81 (15.39) | 52.47 (12.72) | 86.54 (6.64) | 810.08 | < 0.001 | imp < sp; HC > sp; HC > imp |
| **GAF (disability)** |  |  |  |  |  |  |
| lifetime | 77.14 (9.75) | 81.03 (7.63) | 86.73 (5.31) | 87.57 | < 0.001 | imp < sp; HC > sp; HC > imp |
| past year | 63.12 (15.13) | 69.67 (13.1) | 85.64 (6.05) | 223.04 | < 0.001 | imp < sp; HC > sp; HC > imp |
| past month | 47.82 (13.61) | 54.7 (14.44) | 85.14 (6.41) | 654.31 | < 0.001 | imp < sp; HC > sp; HC > imp |
| **BDI** | 19.41 (11.85) | 24.43 (11.83) | 3.25 (4.73) | 323.53 | < 0.001 | imp < sp; HC < imp; HC < sp |
| **PANSS^5^** |  |  |  |  |  |  |
| positive symptoms | 15.02 (6.74) | 11.89 (5.42) | - | 4.73 | < 0.001 | imp > sp |
| negative symptoms | 15.75 (8.03) | 12.76 (5.36) | - | 3.96 | < 0.001 | imp > sp |
| general symptoms | 32.04 (10.28) | 29.43 (8.27) | - | 2.58 | 0.013 | imp > sp |

^Note. 1 = Munich/BASEL/Cologne/Birmingham/Turku/Udine/Milan; 2 = ROP/ROD/CHR; 3 = calculated for ROP and ROD; 4 = cumulative sum of chlorpromazine equivalent divided by number of days treated; calculated for ROP and ROD; 5 = calculated for ROP, ROD and CHR.^

^Abbreviations. imp = impaired ; sp = spared; GAF = global assessment of functioning; BDI = Beck's Depression Inventory; PANSS = positive and negative Syndrome Scale; fdr = false-discovery rate; ROP = recent-onset psychosis patients; ROD = recent-onset depression patients; CHR = clinical high-risk individuals for psychosis; HC = healthy control.^

**Table S4.** Cognitive cluster characteristics of the ROP cluster solution (discovery sample).

|  | **ROP** | | | | | |
| --- | --- | --- | --- | --- | --- | --- |
|  | **impaired (N = 42)** | **spared (N = 98)** | **HC (N = 270)** | **ANOVA** | | |
|  |  |  |  | **F** | **p fdr** | **individual comparisons** |
| **soccog** | -0.28 (1.18) | 0.12 (0.89) | 0.29 (0.64) | 10.28 | < 0.001 | imp < sp; imp < HC; sp = HC |
| **wm** | -2.12 (2.34) | 0.91 (2.12) | 1.64 (1.92) | 63.52 | < 0.001 | imp < sp; imp < HC; sp < HC |
| **proc** | -2.26 (1.54) | 0.97 (1.68) | 1.71 (1.52) | 117.73 | < 0.001 | imp < sp; imp < HC; sp < HC |
| **exfun** | -0.73 (1.28) | 0.31 (0.65) | 0.5 (0.38) | 76.32 | < 0.001 | imp < sp; imp < HC; sp < HC |
| **att** | -2.39 (4.17) | 1.02 (2.26) | 2.25 (2.15) | 67.1 | < 0.001 | imp < sp; imp < HC; sp < HC |
| **verbmem** | -1.34 (1.35) | 0.57 (1.3) | 1.06 (1.0) | 84.88 | < 0.001 | imp < sp; imp < HC; sp < HC |
| **vismem** | -2.44 (2.38) | 1.05 (1.57) | 1.15 (1.38) | 99.55 | < 0.001 | imp < sp; imp < HC; sp = HC |
| **sal** | -0.08 (1.41) | 0.03 (0.96) | -0.14 (0.8) | 1.29 | 0.275 | - |
| **WAIS** |  |  |  |  |  |  |
| vocab | 7.88 (3.43) | 10.66 (3.02) | 11.82 (2.9) | 33.27 | < 0.001 | imp < sp; imp < HC; sp < HC |
| matrix | 7.62 (2.49) | 10.22 (2.3) | 11.14 (2.23) | 45.02 | < 0.001 | imp < sp; imp < HC; sp < HC |

^abbreviations: soccog = social cognition; wm = working memory; proc = processing speed; exfun = executive functioning; att = attention; verbmem = verbal memory; vismem = visual memory; sal = salience; WAIS = Wechsler Adult Intelligence Scale; vocab = vocabulary subtest; matrix = matrix subtest; fdr = false-discovery rate; ROP = recent-onset psychosis patients; HC = healthy controls; sp = spared; imp = impaired.^

**Table S5.** Cognitive cluster characteristics of the ROD cluster solution (discovery sample).

|  | **ROD** | | | | | |
| --- | --- | --- | --- | --- | --- | --- |
|  | **impaired (N = 45)** | **spared (N = 85)** | **HC (N = 270)** | **ANOVA** | | |
|  |  |  |  | **F** | **p fdr** | individual comparisons |
| **soccog** | -0.29 (1.2) | 0.15 (0.85) | 0 (0.86) | 3.59 | 0.032 | imp < sp; HC = sp; HC = imp |
| **wm** | -1.76 (2.04) | 0.93 (2.09) | 0.44 (2.04) | 27.38 | < 0.001 | imp < sp; imp < HC; sp = HC |
| **proc** | -1.78 (1.9) | 0.94 (1.78) | 0.43 (1.54) | 43.85 | < 0.001 | imp < sp; sp > HC; imp < HC |
| **exfun** | -0.75 (1.03) | 0.4 (0.72) | 0.13 (0.75) | 33.03 | < 0.001 | imp < sp; sp > HC; imp < HC |
| **att** | -2.85 (3.69) | 1.51 (2.42) | 0.24 (2.75) | 35.91 | < 0.001 | imp < sp; sp > HC; imp < HC |
| **verbmem** | -1.07 (1.43) | 0.57 (1.25) | 0.23 (1.24) | 26.06 | < 0.001 | imp < sp; sp > HC; imp < HC |
| **vismem** | -1.66 (2.46) | 0.88 (1.87) | 0.26 (1.95) | 24.53 | < 0.001 | imp < sp; sp > HC; imp < HC |
| **sal** | -0.26 (1.11) | 0.14 (1.18) | -0.08 (1.13) | 2.0 | 0.136 | - |
| **WAIS** |  |  |  |  |  |  |
| vocab | 9.87 (2.85) | 11.96 (2.49) | 11.82 (2.9) | 10.06 | < 0.001 | imp < sp; imp < HC; sp = HC |
| matrix | 9.44 (2.25) | 11.6 (2.01) | 11.14 (2.23) | 15.07 | < 0.001 | imp < sp; imp < HC; sp = HC |

^abbreviations: soccog = social cognition; wm = working memory; proc = processing speed; exfun = executive functioning; att = attention; verbmem = verbal memory; vismem = visual memory; sal = salience; WAIS = Wechsler Adult Intelligence Scale; vocab = vocabulary subtest; matrix = matrix subtest; fdr = false-discovery rate; ROD = recent-onset depression patients; HC = healthy controls; sp = spared; imp = impaired.^

**Table S6.** Cognitive cluster characteristics of the CHR cluster solution (discovery sample).

|  | **CHR** | | | | | |
| --- | --- | --- | --- | --- | --- | --- |
|  | **impaired (N = 59)** | **spared (N = 69)** | **HC (N = 270)** | **ANOVA** | | |
|  |  |  |  | **F** | **p fdr** | **individual comparisons** |
| **soccog** | -0.18 (1.05) | 0.15 (0.94) | 0.18 (0.81) | 4.09 | 0.017 | imp < HC |
| **wm** | -1.61 (1.91) | 1.38 (2.11) | 0.22 (2.03) | 35.16 | < 0.001 | imp < sp; sp > HC; imp < HC |
| **proc** | -1.3 (1.7) | 1.11 (1.76) | 0.17 (1.59) | 34.8 | < 0.001 | imp < sp; sp > HC; imp < HC |
| **exfun** | -0.48 (1.23) | 0.41 (0.45) | 0.25 (0.57) | 32.14 | < 0.001 | imp < sp; imp < HC; sp = HC |
| **att** | -1.7 (3.52) | 1.45 (2.72) | 0.48 (2.78) | 20.09 | < 0.001 | imp < sp; imp < HC; sp = HC |
| **verbmem** | -0.94 (1.3) | 0.81 (1.05) | 0.52 (1.19) | 43.28 | < 0.001 | imp < sp; imp < HC; sp = HC |
| **vismem** | -0.77 (2.67) | 0.66 (1.93) | 0.63 (1.68) | 13.74 | < 0.001 | imp < sp; imp < HC; sp = HC |
| **sal** | -0.33 (1.14) | 0.28 (1.04) | 0.08 (0.92) | 6.49 | 0.002 | imp < sp; imp < HC; sp = HC |
| **WAIS** |  |  |  |  |  |  |
| vocab | 10.05 (2.56) | 11.96 (3.04) | 11.82 (2.9) | 9.87 | < 0.001 | imp < sp; imp < HC; sp = HC |
| matrix | 10.07 (2.19) | 11.38 (2.19) | 11.14 (2.23) | 6.75 | 0.002 | imp < sp; imp < HC; sp = HC |

^abbreviations: soccog = social cognition; wm = working memory; proc = processing speed; exfun = executive functioning; att = attention; verbmem = verbal memory; vismem = visual memory; sal = salience; WAIS = Wechsler Adult Intelligence Scale; vocab = vocabulary subtest; matrix = matrix subtest; fdr = false-discovery rate; CHR = clinical high-risk individuals; HC = healthy controls; sp = spared; imp = impaired.^

**Table S7.** Cognitive cluster characteristics across individual clusterings based on ROP, ROD and CHR for discovery and replication sample.

|  | **f_cluster_** | **p_cluster_ fdr** | **f_studygroup_** | **p_studygroup_ fdr** | **f_interaction_** | **p_interaction_ fdr** |
| --- | --- | --- | --- | --- | --- | --- |
| **discovery** |  |  |  |  |  |  |
| soccog | 3.03 | 0.123 | 2.16 | 0.156 | 0.22 | 0.802 |
| wm | 62.16 | < 0.001^1^ | 12.69 | < 0.001^2^ | 0.33 | 0.751 |
| proc | 61.64 | < 0.001^1^ | 26.6 | < 0.001^2^ | 1.97 | 0.177 |
| exfun | 24.84 | < 0.001^1^ | 19.27 | < 0.001^2^ | 4.58 | 0.019^3^ |
| att | 28.61 | < 0.001^1^ | 16.45 | < 0.001^2^ | 1.38 | 0.304 |
| verbmem | 54.26 | < 0.001^1^ | 19.37 | < 0.001^2^ | 2.26 | 0.149 |
| vismem | 14.07 | < 0.001^1^ | 27.27 | < 0.001^2^ | 14.32 | < 0.001^4^ |
| sal | 6.25 | 0.021 | 1.19 | 0.348 | 0.71 | 0.537 |
| **replication** |  |  |  |  |  |  |
| soccog | 4.39 | 0.068 | 1.64 | 0.292 | 0.41 | 0.788 |
| wm | 39.46 | < 0.001^1^ | 5.83 | 0.007^2^ | 0.37 | 0.788 |
| proc | 21.87 | < 0.001^1^ | 11.23 | < 0.001^2^ | 0.57 | 0.716 |
| exfun | 14.69 | < 0.001^1^ | 5.39 | 0.01^5^ | 0.19 | 0.851 |
| att | 22.48 | < 0.001^1^ | 12.5 | < 0.001^2^ | 1.58 | 0.294 |
| verbmem | 28.15 | < 0.001^1^ | 9.45 | < 0.001^2^ | 2.32 | 0.16 |
| vismem | 3.31 | 0.12 | 17.49 | < 0.001 | 7.76 | 0.001^4^ |
| sal | 0.04 | 0.851 | 0.23 | 0.851 | 0.79 | 0.604 |

^Note. 1: imp < sp; 2: ROP < CHR; ROP < ROD; ROD = CHR; 3: impaired subgroups: ROP << ROD, ROP < CHR; spared subgroups: ROP = ROD, ROP = CHR; 4: impaired subgroups: ROP < ROD, ROP < CHR; spared subgroups: ROP = ROD, ROP = CHR; 5: ROP < ROD; ROP = CHR; ROD = CHR.^

^abbreviations: soccog = social cognition; wm = working memory; proc = processing speed; exfun = executive functioning; att = attention; verbmem = verbal memory; vismem = visual memory; sal = salience; WAIS = Wechsler Adult Intelligence Scale; vocab = vocabulary subtest; matrix = matrix subtest; fdr = false-discovery rate; ROP = recent-onset psychosis patients; ROD = recent-onset depression patients; CHR = clinical high-risk individuals; HC = healthy controls.^

**Table S8.** Demographic and clinical characteristics of the ROP cluster solution (discovery sample).

|  | **ROP** | | | | | |
| --- | --- | --- | --- | --- | --- | --- |
|  | **impaired (N = 42)** | **spared (N=98)** | **HC (N=270)** | **ANOVA** | | |
|  |  |  |  | **F/t** | **p fdr** | **individual comparisons** |
| **age** | 25.46 (5.14) | 25.66 (5.7) | 25.4 (6.33) | 0.06 | 0.939 | - |
| **sex, male/female** | 25/17 | 61/37 | 105/165 | 18.91 | < 0.001 | HC: female > male |
| **site^1^** | 10/3/9/2/8/3/7 | 31/17/16/6/15/6/7 | 58/39/59/43/23/35/13 | 31.08 | 0.003 | Milan: imp ~ sp; others: imp < sp |
| **studygroup^2^** | 42/0/0 | 98/0/0 | - | - | - |  |
| **years in education** | 13.73 (2.98) | 14.23 (3.08) | 15.68 (3.22) | 12.2 | < 0.001 | HC > imp; HC > sp |
| **illness duration, days^3^** | 190.63 (167.71) | 176.97 (180.61) | - | 0.43 | 0.763 | - |
| **CPZ^4^** | 746.92 (1580.06) | 238.64 (372.09) | - | 1.71 | 0.130 | - |
| **GAF (symptoms)** |  |  |  |  |  |  |
| lifetime | 75.4 (10.99) | 80.2 (9.57) | 88.04 (5.69) | 77.28 | < 0.001 | imp < sp; HC > sp; HC > imp |
| past year | 57.19 (15.3) | 61.4 (17.09) | 86.97 (6.26) | 279.01 | < 0.001 | imp < sp; HC > sp; HC > imp |
| past month | 38.17 (13.65) | 42.59 (13.12) | 86.54 (6.64) | 1056.14 | < 0.001 | imp < sp; HC > sp; HC > imp |
| **GAF (disability)** |  |  |  |  |  |  |
| lifetime | 73.83 (9.21) | 80 (8.73) | 86.73 (5.31) | 86.71 | < 0.001 | imp < sp; HC > sp; HC > imp |
| past year | 58.19 (14.16) | 64.4 (14.77) | 85.64 (6.05) | 259.63 | < 0.001 | imp < sp; HC > sp; HC > imp |
| past month | 41.4 (10.19) | 46.65 (12.53) | 85.14 (6.41) | 983.99 | < 0.001 | imp < sp; HC > sp; HC > imp |
| **BDI** | 18.23 (11.86) | 20.43 (11.25) | 3.25 (4.73) | 203.49 | < 0.001 | imp < sp; HC < imp; HC < sp |
| **PANSS** |  |  |  |  |  |  |
| positive symptoms | 19.12 (6.94) | 18.77 (5.59) | - | 0.29 | 0.827 | - |
| negative symptoms | 19.44 (7.82) | 15.1 (7.5) | - | 3.01 | 0.005 | imp > sp |
| general symptoms | 36.68 (11.08) | 34.92 (10.5) | - | 0.87 | 0.478 | - |

^Note. 1 = Munich/BASEL/Cologne/Birmingham/Turku/Udine/Milan; 2 = ROP/ROD/CHR; 3 = cumulative sum of chlorpromazine equivalent divided by number of days treated.^

^Abbreviations. imp = impaired ; sp = spared; GAF = global assessment of functioning; BDI = Beck's Depression Inventory; PANSS = positive and negative Syndrome Scale; fdr = false-discovery rate; HC = healthy control; ROP = recent-onset psyhcosis patients.^

**Table S9.** Demographic and clinical characteristics of the ROD cluster solution (discovery sample).

|  | **ROD** | | | | | |
| --- | --- | --- | --- | --- | --- | --- |
|  | **impaired (N=35)** | **spared (N=85)** | **HC (N=270)** | **ANOVA** | | |
|  |  |  |  | **F/t** | **p fdr** | **individual comparisons** |
| **age** | 25.75 (6) | 26.3 (6.19) | 25.4 (6.33) | 0.67 | 0.583 | - |
| **sex, male/female** | 19/26 | 38/47 | 105/165 | 0.97 | 0.657 | - |
| **site^1^** | 11/7/9/2/4/9/3 | 35/8/13/10/7/8/4 | 58/39/59/43/23/35/13 | 19.28 | 0.131 | - |
| **studygroup^2^** | 0/45/0 | 0/85/0 | - | - | - |  |
| **years in education** | 14.28 (3.18) | 15.1 (3.02) | 15.68 (3.22) | 4.24 | 0.030 | HC > imp |
| **illness duration, days** | 238.47 (166.53) | 227.53 (212.88) | - | 0.32 | 0.748 | - |
| **CPZ^3^** | 52.44 (38.81) | 116.31 (150.13) | - | -1.47 | 0.237 | - |
| **GAF (symptoms)** |  |  |  |  |  |  |
| lifetime | 81.36 (7.98) | 82.64 (6.63) | 88.04 (5.69) | 39.4 | < 0.001 | HC > sp; HC > imp |
| past year | 68.73 (14.71) | 73.27 (10.26) | 86.97 (6.26) | 143.52 | < 0.001 | imp < sp; HC > sp; HC > imp |
| past month | 55.7 (12.86) | 55.99 (12.29) | 86.54 (6.64) | 510.38 | < 0.001 | HC > sp; HC > imp |
| **GAF (disability)** |  |  |  |  |  |  |
| lifetime | 79.7 (8.87) | 81.48 (7.97) | 86.73 (5.31) | 37.26 | < 0.001 | HC > sp; HC > imp |
| past year | 67.18 (16.47) | 72.89 (11.79) | 85.64 (6.05) | 117.52 | < 0.001 | imp < sp; HC > sp; HC > imp |
| past month | 52.8 (15.28) | 58.15 (14.37) | 85.14 (6.41) | 375.65 | < 0.001 | imp < sp; HC > sp; HC > imp |
| **BDI** | 20.56 (12.27) | 25.73 (12.61) | 3.25 (4.73) | 281.62 | < 0.001 | imp < sp; HC < imp; HC < sp |
| **PANSS** |  |  |  |  |  |  |
| positive symptoms | 8.75 (2.37) | 8.04 (1.25) | - | 1.86 | 0.121 | - |
| negative symptoms | 13.14 (5.16) | 12.14 (4.71) | - | 1.06 | 0.389 | - |
| general symptoms | 28.09 (7) | 26.94 (6.53) | - | 0.9 | 0.455 | - |

^Note. 1 = Munich/BASEL/Cologne/Birmingham/Turku/Udine/Milan; 2 = ROP/ROD/CHR; 3 = cumulative sum of chlorpromazine equivalent divided by number of days treated.^

^Abbreviations. imp = impaired ; sp = spared; GAF = global assessment of functioning; BDI = Beck's Depression Inventory; PANSS = positive and negative Syndrome Scale; fdr = false-discovery rate; HC = healthy control; ROP = recent-onset psychosis patients; ROD = recent-onset depression patients: CHR = clinical high-risk individuals.^

**Table S10.** Demographic and clinical characteristics of the CHR cluster solution (discovery sample).

|  | **CHR** | | | | | |
| --- | --- | --- | --- | --- | --- | --- |
|  | **impaired (N=59)** | **spared (N=69)** | **HC (N=270)** | **ANOVA** | | |
|  |  |  |  | **F/t** | **p fdr** | **individual comparisons** |
| **age** | 24.64 (5.32) | 22.96 (4.68) | 25.4 (6.33) | 4.70 | 0.015 | sp < HC |
| **sex, male/female** | 32/27 | 35/34 | 105/165 | 6.57 | 0.053 | - |
| **site^1^** | 15/10/11/4/8/5/6 | 26/7/8/9/7/11/1 | 58/39/59/43/23/35/13 | 20.47 | 0.075 | - |
| **studygroup^2^** | 0/0/59 | 0/0/69 | - | - | - |  |
| **years in education** | 14.08 (3.47) | 13.28 (2.5) | 15.68 (3.22) | 19.08 | < 0.001 | HC > sp; HC > imp |
| **illness duration, days** | - | - | - | - | - | - |
| **CPZ^3^** | - | - | - | - | - | - |
| **GAF (symptoms)** |  |  |  |  |  |  |
| lifetime | 78.86 (8.4) | 80.74 (8.81) | 88.04 (5.69) | 64.18 | < 0.001 | HC > sp; HC > imp |
| past year | 65.36 (12.53) | 69.51 (11.59) | 86.97 (6.26) | 226.25 | < 0.001 | imp < sp; HC > sp; HC > imp |
| past month | 54.56 (11.69) | 54.94 (10.2) | 86.54 (6.64) | 642.17 | < 0.001 | HC > sp; HC > imp |
| **GAF (disability)** |  |  |  |  |  |  |
| lifetime | 79.05 (8.89) | 80.71 (7.37) | 86.73 (5.31) | 50.58 | < 0.001 | HC > sp; HC > imp |
| past year | 65.46 (13.75) | 71.55 (10.79) | 85.64 (6.05) | 177.76 | < 0.001 | imp < sp; HC > sp; HC > imp |
| past month | 53.15 (12.53) | 57.91 (14.41) | 85.14 (6.41) | 435.06 | < 0.001 | imp < sp; HC > sp; HC > imp |
| **BDI** | 22.95 (11.21) | 25.05 (12.06) | 3.25 (4.73) | 306.81 | < 0.001 | HC < imp; HC < sp |
| **PANSS** |  |  |  |  |  |  |
| positive symptoms | 11.57 (3.28) | 11.38 (3.49) | - | 0.32 | 0.750 | - |
| negative symptoms | 12.86 (6.55) | 12.1 (5.21) | - | 0.71 | 0.556 | - |
| general symptoms | 27.6 (7.07) | 28.23 (6.7) | - | -0.51 | 0.655 | - |

^Note. 1 = Munich/BASEL/Cologne/Birmingham/Turku/Udine/Milan; 2 = ROP/ROD/CHR; 3 = cumulative sum of chlorpromazine equivalent divided by number of days treated, not calculated for CHR.^

^Abbreviations. imp = impaired ; sp = spared; GAF = global assessment of functioning; BDI = Beck's Depression Inventory; PANSS = positive and negative Syndrome Scale; fdr = false-discovery rate; HC = healthy control; ROP = recent-onset psychosis patients; ROD = recent-onset depression patients: CHR = clinical high-risk individuals.^

**Table S11.** Supervised classifier performances for classification of individual study groups against healthy controls.

|  | **classifier performance** | | | | | |
| --- | --- | --- | --- | --- | --- | --- |
|  | **Sens** | **Spec** | **BAC** | **PPV** | **NPV** | **model p value** |
| **GMV** |  |  |  |  |  |  |
| ROP vs HC | 38.8 | 59.8 | 49.3 | 34.0 | 64.7 | p = 0.58 |
| ROD vs HC | 44.9 | 60.9 | 52.9 | 35.8 | 69.4 | p = 0.15 |
| CHR vs HC | 39.7 | 67.0 | 53.4 | 36.8 | 69.7 | p = 0.09 |
| **rsFC** |  |  |  |  |  |  |
| ROP vs HC | 61.9 | 68.2 | 65.0 | 50.9 | 77.1 | p < 0.01 |
| ROD vs HC | 49.6 | 67.0 | 58.3 | 42.3 | 73.2 | p < 0.01 |
| CHR vs HC | 42.9 | 75.5 | 59.2 | 45.8 | 73.2 | p < 0.01 |

^Abbreviations: sens = sensitivity; spec = specificity; BAC = balanced accuracy; PPV = positive predictive value; NPV = negative predictive value; GMV = grey matter volume; rsFC = resting-state functional connectivity; ROP = recent-onset psychosis individuals; ROD = recent-onset depression individuals; CHR = clinical high-risk individuals; HC = healthy controls.^

**Table S12.** Most predictive rsFC features in the 'impaired vs HC'- and 'spared vs HC'- model. Positive CV ratios indicate prediction of impaired (left column) or spared (right column) subgroup and negative CV ratios indicate prediction of HC. The ten most predictive features for each group are displayed.

| **impaired vs HC model** | | **spared vs HC model** | |
| --- | --- | --- | --- |
| **predictive features** | **CV ratio (grand mean)** | **predictive features** | **CV ratio (grand mean)** |
| occipital_-9_-72_41_IPL_-41_-40_42 | 5.40534 | occipital_15_-77_32_IPS_-32_-58_46 | 5.54046 |
| inf temporal_-61_-41_-2_thalamus_11_-12_6 | 5.28466 | occipital_-28_-42_-11_post cingulate_-4_-31_-4 | 5.41004 |
| inf temporal_-61_-41_-2_thalamus_-12_-3_13 | 5.27145 | occipital_-9_-72_41_angular gyrus_-41_-47_29 | 5.38899 |
| occipital_-2_-75_32_IPS_-32_-58_46 | 5.25244 | occipital_-16_-76_33_occipital_-29_-75_28 | 5.3608 |
| occipital_-9_-72_41_post parietal_-35_-46_48 | 5.22405 | occipital_15_-77_32_occipital_-29_-75_28 | 5.33923 |
| basal ganglia_11_-24_2_parietal_-47_-12_36 | 5.18079 | precuneus_11_-68_42_IPS_-32_-58_46 | 5.32591 |
| occipital_-9_-72_41_post parietal_-41_-31_48 | 5.17869 | occipital_-9_-72_41_IPS_-32_-58_46 | 5.29227 |
| inf cerebellum_-6_-79_-33_post parietal_-41_-31_48 | 5.15677 | occipital_15_-77_32_IPS_32_-59_41 | 5.28095 |
| post occipital_27_-91_2_thalamus_11_-12_6 | 5.15554 | occipital_17_-68_20_IPS_32_-59_41 | 5.26407 |
| basal ganglia_11_-24_2_mid insula_-42_-3_11 | 5.13924 | post parietal_-35_-46_48_precuneus_8_-40_50 | 5.25558 |
| inf cerebellum_-37_-54_-37_thalamus_11_-12_6 | -4.37410 | IPS_-36_-69_40_dlPFC_40_36_29 | -4.98181 |
| post cingulate_-4_-31_-4_thalamus_-12_-12_6 | -4.42004 | thalamus_-12_-12_6_aPFC_29_57_18 | -4.9943 |
| inf temporal_-59_-25_-15_dFC_-42_7_36 | -4.43414 | inf temporal_-59_-25_-15_vFC_51_23_8 | -5.0061 |
| angular gyrus_-48_-63_35_aPFC_-25_51_27 | -4.43901 | occipital_15_-77_32_vmPFC_8_42_-5 | -5.01298 |
| inf temporal_52_-15_-13_precentral gyrus_-54_-9_23 | -4.44983 | IPL_44_-52_47_sup frontal_-16_29_54 | -5.01571 |
| IPS_-36_-69_40_ACC_-1_28_40 | -4.50156 | IPS_-36_-69_40_vent aPFC_42_48_-3 | -5.06797 |
| inf cerebellum_-6_-79_-33_thalamus_11_-12_6 | -4.61793 | IPL_54_-44_43_sup frontal_-16_29_54 | -5.1099 |
| inf cerebellum_33_-73_-30_thalamus_-12_-12_6 | -4.64641 | inf temporal_-59_-25_-15_mid insula_-36_-12_15 | -5.22409 |
| inf cerebellum_33_-73_-30_thalamus_11_-12_6 | -4.72928 | parietal_-55_-44_30_vlPFC_46_39_-15 | -5.38664 |
| inf cerebellum_-21_-79_-33_thalamus_11_-12_6 | -4.77933 | angular gyrus_-48_-63_35_dlPFC_46_28_31 | -5.46676 |

^Abbreviation: CV ratio = cross-validation ratio.^

**Table S13.** Demographic and clinical characteristics of the transdiagnostic cluster solution (replication sample).

|  | **transdiagnostic** | | | | | |
| --- | --- | --- | --- | --- | --- | --- |
|  | **impaired (N = 161)** | **spared (N = 128)** | **HC (N = 120)** | **ANOVA** | | |
|  |  |  |  | **F/t** | **p fdr** | **individual comparisons** |
| **age in years** | 25.44 (6.15) | 23.69 (5.82) | 24.42 (5.28) | 3.32 | 0.053 | - |
| **sex, male/female** | 77/84 | 63/65 | 54/66 | 0.46 | 0.795 | - |
| **site^1^** | 82/4/17/11/19/6/22 | 55/6/37/7/7/3/13 | 6/23/11/6/30/35/9 | 165.7 | < 0.001 | Basel: imp ~ sp; Cologne: sp > imp; others: imp > sp |
| **studygroup^2^** | 81/32/48 | 27/49/52 | - | 27.32 | < 0.001 | imp: ROP > ROD/CHR; sp: ROD > ROP/CHR |
| **years in education** | 14.43 (3.14) | 13.12 (2.36) | 15.17 (3.14) | 15.91 | < 0.001 | sp < imp; HC > sp; HC > imp |
| **illness duration, days^3^** | 207.93 (188.45) | 274.07 (243.13) | - | -2.00 | 0.062 | - |
| **CPZ^4^** | 327.34 (607.11) | 178.88 (136.28) | - | 1.72 | 0.109 | - |
| **GAF (symptoms)** |  |  |  |  |  |  |
| lifetime | 77.99 (9.41) | 77.77 (8.82) | 87.6 (5.01) | 59.86 | < 0.001 | HC > sp; HC > imp |
| past year | 63.17 (14.94) | 60.94 (11.46) | 86.64 (5.6) | 185.02 | < 0.001 | HC > sp; HC > imp |
| past month | 45.11 (14.18) | 49.65 (12.26) | 86.38 (5.6) | 486.93 | < 0.001 | imp < sp; HC > sp; HC > imp |
| **GAF (disability)** |  |  |  |  |  |  |
| lifetime | 78.85 (8.67) | 78.21 (8.84) | 87.6 (4.27) | 58.32 | < 0.001 | HC > sp; HC > imp |
| past year | 64.1 (14.73) | 63.6 (12.58) | 86.58 (4.88) | 154.04 | < 0.001 | HC > sp; HC > imp |
| past month | 47.1 (13.98) | 50.54 (13.63) | 86.38 (4.83) | 427.91 | < 0.001 | imp < sp; HC > sp; HC > imp |
| **BDI** | 21.67 (11.68) | 24.59 (12.57) | 3.57 (5.55) | 128.66 | < 0.001 | imp < sp; HC < imp; HC < sp |
| **PANSS^5^** |  |  |  |  |  |  |
| positive symptoms | 15.65 (6.83) | 12.29 (5.24) | - | 4.60 | < 0.001 | imp > sp |
| negative symptoms | 15.04 (6.78) | 14.4 (6.26) | - | 0.81 | 0.447 | - |
| general symptoms | 32.68 (9.64) | 31.02 (8.14) | - | 1.53 | 0.145 | - |

^Note. 1 = Munich/BASEL/Cologne/Birmingham/Turku/Udine/Milan; 2 = ROP/ROD/CHR; 3 = calculated for ROP and ROD; 4 = cumulative sum of chlorpromazine equivalent divided by number of days treated; calculated for ROP and ROD; 5 = calculated for ROP, ROD and CHR.^

^Abbreviations. imp = impaired ; sp = spared; GAF = global assessment of functioning; BDI = Beck's Depression Inventory; PANSS = positive and negative Syndrome Scale; fdr = false-discovery rate; ROP = recent-onset psychosis patients; ROD = recent-onset depression patients; CHR = clinical high-risk individuals for psychosis; HC = healthy control.^

**Table S14.** Cognitive cluster characteristics of the ROP cluster solution (replication sample).

|  | **ROP** | | | | | |
| --- | --- | --- | --- | --- | --- | --- |
|  | **impaired (N = 56)** | **spared (N = 52)** | **HC (N = 120)** | **ANOVA** | | |
|  |  |  |  | **F** | **p fdr** | **individual comparisons** |
| **soccog** | -0.61 (1.49) | 0.52 (2.21) | 0.29 (0.7) | 10.89 | < 0.001 | imp < sp; sp = HC; imp < HC |
| **wm** | -1.7 (2.48) | 1.44 (3.27) | 1.64 (1.37) | 46.2 | < 0.001 | imp < sp; sp = HC; imp < HC |
| **proc** | -3.18 (3.98) | 0.4 (3.15) | 1.71 (1.4) | 63.89 | < 0.001 | imp < sp; sp < HC; imp < HC |
| **exfun** | -0.57 (0.94) | 0.18 (0.76) | 0.5 (0.34) | 53.73 | < 0.001 | imp < sp; sp < HC; imp < HC |
| **att** | -5.11 (6.46) | 1.45 (3.44) | 2.25 (1.75) | 74.59 | < 0.001 | imp < sp; sp = HC; imp < HC |
| **verbmem** | -0.34 (1.36) | 1.09 (1.41) | 1.06 (0.91) | 31.22 | < 0.001 | imp < sp; sp = HC; imp < HC |
| **vismem** | -2.37 (3.06) | 1.11 (2.15) | 1.15 (1.33) | 60.88 | < 0.001 | imp < sp; sp = HC; imp < HC |
| **sal** | -0.03 (2.03) | -0.8 (2.77) | -0.14 (0.79) | 3.22 | 0.042 | sp < imp; HC = sp; imp = HC |
| **WAIS** |  |  |  |  |  |  |
| vocab | 8.89 (3.45) | 10.21 (2.85) | 12.65 (2.54) | 36.76 | < 0.001 | imp < sp; sp < HC; imp < HC |
| matrix | 9.02 (3.04) | 10.42 (2.4) | 11.94 (1.97) | 30.33 | < 0.001 | imp < sp; sp < HC; imp < HC |

^abbreviations: soccog = social cognition; wm = working memory; proc = processing speed; exfun = executive functioning; att = attention; verbmem = verbal memory; vismem = visual memory; sal = salience; WAIS = Wechsler Adult Intelligence Scale; vocab = vocabulary subtest; matrix = matrix subtest; fdr = false-discovery rate; ROP = recent-onset psychosis patients; HC = healthy controls; sp = spared; imp = impaired.^

**Table S15.** Cognitive cluster characteristics of the ROD cluster solution (replication sample).

|  | **ROD** | | | | | |
| --- | --- | --- | --- | --- | --- | --- |
|  | **impaired (N = 42)** | **spared (N = 39)** | **HC (N = 120)** | **ANOVA** | |  |
|  |  |  |  | **F** | **p fdr** | **individual comparisons** |
| **soccog** | -0.42 (1.96) | 0.95 (1.92) | 0 (0.94) | 10.0 | < 0.001 | imp < sp; sp > HC; imp < HC |
| **wm** | -1.08 (2.73) | 1.72 (2.66) | 0.44 (1.44) | 19.23 | < 0.001 | imp < sp; sp > HC; imp < HC |
| **proc** | -2.69 (2.55) | 0.32 (2.48) | 0.43 (1.43) | 42.37 | < 0.001 | imp < sp; sp = HC; imp < HC |
| **exfun** | -0.98 (1.25) | 0.23 (1.21) | 0.13 (0.68) | 24.21 | < 0.001 | imp < sp; sp = HC; imp < HC |
| **att** | -5.4 (6.74) | 2.78 (4.38) | 0.24 (2.29) | 46.0 | < 0.001 | imp < sp; sp > HC; imp < HC |
| **verbmem** | -0.26 (1.41) | 0.63 (1.54) | 0.23 (1.14) | 4.88 | 0.009 | imp < sp; sp = HC; imp = HC |
| **vismem** | -1.3 (3.44) | 0.69 (2.51) | 0.26 (1.93) | 8.25 | < 0.001 | imp < sp; sp = HC; imp < HC |
| **sal** | -1.03 (1.71) | -0.79 (1.78) | -0.08 (1.02) | 9.49 | < 0.001 | imp < sp; sp < HC; imp < HC |
| **WAIS** |  |  |  |  |  |  |
| vocab | 10.45 (3.09) | 11.28 (2.29) | 12.65 (2.54) | 12.4 | < 0.001 | imp = sp; imp < HC; sp < HC |
| matrix | 10.38 (2.21) | 11.56 (2.61) | 11.94 (1.97) | 8.16 | < 0.001 | imp < sp; sp = HC; imp < HC |

^abbreviations: soccog = social cognition; wm = working memory; proc = processing speed; exfun = executive functioning; att = attention; verbmem = verbal memory; vismem = visual memory; sal = salience; WAIS = Wechsler Adult Intelligence Scale; vocab = vocabulary subtest; matrix = matrix subtest; fdr = false-discovery rate; ROD = recent-onset depression patients; HC = healthy controls; sp = spared; imp = impaired.^

**Table S16.** Cognitive cluster characteristics of the CHR cluster solution (replication sample).

|  | **CHR** | | | | | |
| --- | --- | --- | --- | --- | --- | --- |
|  | **impaired (N = 63)** | **spared (N = 37)** | **HC (N = 120)** | **ANOVA** | | |
|  |  |  |  | **F** | **p fdr** | **individual comparisons** |
| **soccog** | -0.45 (2.04) | 0.45 (1.81) | 0.18 (0.88) | 5.52 | 0.006 | imp < sp; imp < HC; sp = HC |
| **wm** | -1.95 (2.29) | 1.54 (2.05) | 0.22 (1.43) | 49.07 | < 0.001 | imp < sp; sp > HC; imp < HC |
| **proc** | -2.31 (2.45) | 0.47 (2.14) | 0.17 (1.45) | 40.7 | < 0.001 | imp < sp; imp < HC; sp = HC |
| **exfun** | -0.79 (1.5) | 0.2 (1.19) | 0.25 (0.51) | 23.13 | < 0.001 | imp < sp; imp < HC; sp = HC |
| **att** | -3.8 (5.47) | 1.99 (3.89) | 0.48 (2.34) | 36.61 | < 0.001 | imp < sp; sp > HC; imp < HC |
| **verbmem** | -0.39 (1.76) | 1.37 (1.45) | 0.52 (1.09) | 20.14 | 0.009 | imp < sp; sp > HC; imp < HC |
| **vismem** | -0.69 (3.17) | 0.42 (2.65) | 0.63 (1.63) | 6.75 | 0.002 | imp < sp; imp < HC; sp = HC |
| **sal** | -0.95 (1.54) | -0.27 (1.42) | 0.08 (0.87) | 15.45 | < 0.001 | imp < sp; imp < HC; sp = HC |
| **WAIS** |  |  |  |  |  |  |
| vocab | 10.87 (5.79) | 11.38 (2.86) | 12.65 (2.54) | 4.96 | 0.009 | imp = sp; imp < HC; sp = HC |
| matrix | 10.81 (6.32) | 11.3 (2.7) | 11.94 (1.97) | 1.87 | 0.157 | - |

^abbreviations: soccog = social cognition; wm = working memory; proc = processing speed; exfun = executive functioning; att = attention; verbmem = verbal memory; vismem = visual memory; sal = salience; WAIS = Wechsler Adult Intelligence Scale; vocab = vocabulary subtest; matrix = matrix subtest; fdr = false-discovery rate; CHR = clinical high-risk individuals; HC = healthy controls; sp = spared; imp = impaired.^

**Table S17.** Demographic and clinical characteristics of the ROP cluster solution (replication sample).

|  | **ROP** | | | | | |
| --- | --- | --- | --- | --- | --- | --- |
|  | **impaired (N = 56)** | **spared (N= 52)** | **HC (N= 120)** | **ANOVA** | | |
|  |  |  |  | **F/t** | **p fdr** | **individual comparisons** |
| **age in years** | 25.41 (6.52) | 25.39 (6.18) | 24.42 (5.28) | 0.81 | 0.549 | - |
| **sex, male/female** | 25/31 | 32/20 | 54/66 | 4.46 | 0.157 | - |
| **site^1^** | 33/1/4/5/4/2/7 | 22/2/10/3/10/1/4 | 6/23/11/6/30/35/9 | 97.68 | < 0.001 | Basel/Cologne/Turku: imp > sp; others: spared > imp |
| **studygroup^2^** | 56/0/0 | 52/0/0 | - | - | - |  |
| **years in education** | 14.79 (3.67) | 12.97 (2.64) | 15.17 (3.14) | 8.87 | < 0.001 | sp < imp; sp < HC |
| **illness duration, days** | 228.12 (203.56) | 221.15 (228.57) | - | 0.17 | 0.884 | - |
| **CPZ^3^** | 308.62 (606.6) | 328.36 (480.54) | - | -0.15 | 0.884 | - |
| **GAF (symptoms)** |  |  |  |  |  |  |
| lifetime | 77.2 (8.78) | 77.71 (10.79) | 87.6 (5.01) | 50.16 | < 0.001 | HC > sp; HC > imp |
| past year | 59.88 (15.83) | 59.24 (15.86) | 86.64 (5.6) | 154.41 | < 0.001 | HC > sp; HC > imp |
| past month | 39.64 (13.65) | 42.57 (14.92) | 86.38 (5.6) | 519.75 | < 0.001 | HC > sp; HC > imp |
| **GAF (disability)** |  |  |  |  |  |  |
| lifetime | 77.64 (9.94) | 78.71 (8.59) | 87.6 (4.27) | 50.4 | < 0.001 | HC > sp; HC > imp |
| past year | 62.66 (14.76) | 64.35 (12.77) | 86.58 (4.88) | 147.71 | < 0.001 | HC > sp; HC > imp |
| past month | 42.71 (13.79) | 45.96 (15.19) | 86.38 (4.83) | 454.7 | < 0.001 | HC > sp; HC > imp |
| **BDI** | 19.4 (12.02) | 17.17 (11.56) | 3.57 (5.55) | 67.27 | < 0.001 | HC > sp; HC > imp |
| **PANSS** |  |  |  |  |  |  |
| positive symptoms | 19.89 (5.91) | 19.72 (6.02) | - | 0.15 | 0.884 | - |
| negative symptoms | 16.48 (6.81) | 13.66 (6.02) | - | 2.24 | 0.043 | imp > sp |
| general symptoms | 34.94 (8.52) | 32.14 (9.84) | - | 1.55 | 0.166 | - |

^Note. 1 = Munich/BASEL/Cologne/Birmingham/Turku/Udine/Milan; 2 = ROP/ROD/CHR; 3 = cumulative sum of chlorpromazine equivalent divided by number of days treated.^

^Abbreviations. imp = impaired ; sp = spared; GAF = global assessment of functioning; BDI = Beck's Depression Inventory; PANSS = positive and negative Syndrome Scale; fdr = false-discovery rate; HC = healthy control; ROP = recent-onset psychosis patients; ROD = recent-onset depression patients; CHR = clinical high-risk individuals.^

**Table S18.** Demographic and clinical characteristics of the ROD cluster solution (replication sample).

|  | **ROD** | | | | | |
| --- | --- | --- | --- | --- | --- | --- |
|  | **impaired (N = 42)** | **spared (N = 39)** | **HC (N = 120)** | **ANOVA** | | |
|  |  |  |  | **F/t** | **p fdr** | **individual comparisons** |
| **age in years** | 25.61 (5.74) | 23.05 (5.2) | 24.42 (5.28) | 2.31 | 0.151 | - |
| **sex, male/female** | 26/16 | 16/23 | 54/66 | 4.44 | 0.151 | - |
| **site^1^** | 23/0/10/2/2/0/5 | 11/2/18/2/1/1/4 | 6/23/11/6/30/35/9 | 108.27 | < 0.001 | Munich/Turku/Milan: imp > sp; Birm: imp = sp; others: sp > imp |
| **studygroup^2^** | 0/42/0 | 0/39/0 | - | - | - |  |
| **years in education** | 14.85 (3) | 13.56 (2.55) | 15.17 (3.14) | 4.22 | 0.029 | HC > sp; HC > imp |
| **illness duration, days** | 213.9 (181.11) | 283.74 (240.48) | - | -1.47 | 0.180 | - |
| **CPZ^3^** | 84.5 (43.93) | 57.05 (64.82) | - | 0.78 | 0.524 | - |
| **GAF (symptoms)** |  |  |  |  |  |  |
| lifetime | 80.49 (6.12) | 78.18 (8.7) | 87.6 (5.01) | 44.7 | < 0.001 | HC > sp; HC > imp |
| past year | 68.83 (10.5) | 65.16 (9.79) | 86.64 (5.6) | 155.33 | < 0.001 | imp > sp; HC > sp; HC > imp |
| past month | 53.32 (12.68) | 52.13 (11.96) | 86.38 (5.6) | 341.95 | < 0.001 | HC > sp; HC > imp |
| **GAF (disability)** |  |  |  |  |  |  |
| lifetime | 80.44 (6.88) | 79.68 (7.76) | 87.6 (4.27) | 42.21 | < 0.001 | HC > sp; HC > imp |
| past year | 68.63 (12.8) | 68.21 (11.82) | 86.58 (4.88) | 105.75 | < 0.001 | HC > sp; HC > imp |
| past month | 53.78 (14.78) | 54.55 (14.74) | 86.38 (4.83) | 248 | < 0.001 | HC > sp; HC > imp |
| **BDI** | 25.3 (10.71) | 26.18 (13.3) | 3.57 (5.55) | 138.38 | < 0.001 | HC > sp; HC > imp |
| **PANSS** |  |  |  |  |  |  |
| positive symptoms | 8.22 (1.27) | 8.81 (1.88) | - | -1.61 | 0.151 | - |
| negative symptoms | 13.98 (6.16) | 13.59 (4.95) | - | 0.3 | 0.763 | - |
| general symptoms | 29.65 (8.42) | 30.32 (7.88) | - | -0.36 | 0.763 | - |

^Note. 1 = Munich/BASEL/Cologne/Birmingham/Turku/Udine/Milan; 2 = ROP/ROD/CHR; 3 = cumulative sum of chlorpromazine equivalent divided by number of days treated.^

^Abbreviations. imp = impaired ; sp = spared; GAF = global assessment of functioning; BDI = Beck's Depression Inventory; PANSS = positive and negative Syndrome Scale; fdr = false-discovery rate; HC = healthy control; ROP = recent-onset psychosis patients; ROD = recent-onset depression patients; CHR = clinical high-risk individuals.^

**Table S19.** Demographic and clinical characteristics of the CHR cluster solution (replication sample).

|  | **CHR** | | | | | |
| --- | --- | --- | --- | --- | --- | --- |
|  | **impaired (N = 63)** | **spared (N = 37)** | **HC (N = 120)** | **ANOVA** | | |
|  |  |  |  | **F/t** | **p fdr** | **individual comparisons** |
| **age in years** | 24.12 (6.15) | 24.06 (6.04) | 24.42 (5.28) | 0.09 | 0.914 | - |
| **sex, male/female** | 28/35 | 13/24 | 54/66 | 1.18 | 0.647 | - |
| **site^1^** | 30/3/4/4/7/4/11 | 18/2/8/2/2/1/4 | 6/23/11/6/30/35/9 | 85.22 | < 0.001 | Cologne: sp > imp; others: imp > sp |
| **studygroup^2^** | 0/0/63 | 0/0/37 | - | - | - |  |
| **years in education** | 13.63 (2.28) | 13.2 (2.48) | 15.17 (3.14) | 10.23 | < 0.001 | HC > sp; HC > imp |
| **illness duration, days** | - | - | - |  |  |  |
| **CPZ^3^** | - | - | - |  |  |  |
| **GAF (symptoms)** |  |  |  |  |  |  |
| lifetime | 77.07 (10.29) | 77.36 (8.44) | 87.6 (5.01) | 52.63 | < 0.001 | HC > sp; HC > imp |
| past year | 62.78 (13.17) | 58.17 (9.71) | 86.64 (5.6) | 218.39 | < 0.001 | imp > sp; HC > sp; HC > imp |
| past month | 49.75 (11) | 48.61 (10.62) | 86.38 (5.6) | 526.59 | < 0.001 | HC > sp; HC > imp |
| **GAF (disability)** |  |  |  |  |  |  |
| lifetime | 78.6 (8.99) | 76.42 (9.33) | 87.6 (4.27) | 56.03 | < 0.001 | HC > sp; HC > imp |
| past year | 61.27 (15.47) | 59.42 (11.47) | 86.58 (4.88) | 178.72 | < 0.001 | HC > sp; HC > imp |
| past month | 49.05 (10.67) | 48.83 (10.8) | 86.38 (4.83) | 584.99 | < 0.001 | HC > sp; HC > imp |
| **BDI** | 25.53 (11.46) | 26.06 (11.32) | 3.57 (5.55) | 155.55 | < 0.001 |  |
| **PANSS** |  |  |  |  |  |  |
| positive symptoms | 12.58 (3.59) | 12.31 (3.58) | - | 0.35 | 0.787 | - |
| negative symptoms | 14.75 (7.31) | 15.8 (7.16) | - | -0.67 | 0.639 | - |
| general symptoms | 31.11 (10.06) | 32.6 (7.83) | - | -0.78 | 0.611 | - |

^Note. 1 = Munich/BASEL/Cologne/Birmingham/Turku/Udine/Milan; 2 = ROP/ROD/CHR; 3 = cumulative sum of chlorpromazine equivalent divided by number of days treated.^

^Abbreviations. imp = impaired ; sp = spared; GAF = global assessment of functioning; BDI = Beck's Depression Inventory; PANSS = positive and negative Syndrome Scale; fdr = false-discovery rate; HC = healthy control; ROP = recent-onset psychosis patients; ROD = recent-onset depression patients; CHR = clinical high-risk individuals.^

# Figures

**
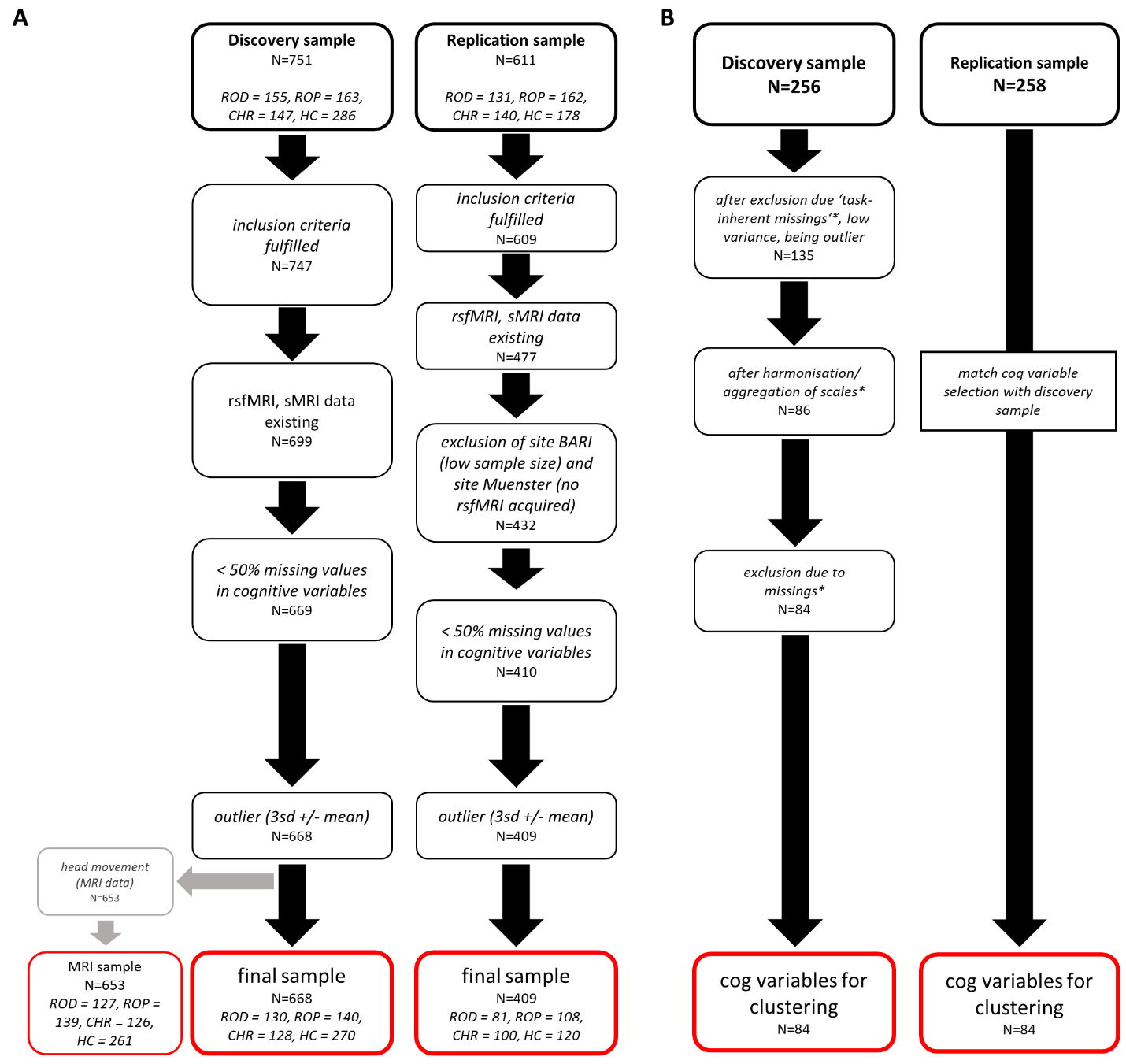
**

**Figure S1.** Flowchart on sample and cognitive variable selection and exclusion. The data selection process is indicated for (A) participants and (B) cognitive variables both in discovery and replication data set. * Aggregations and transformations conducted on the salience and Rey auditory verbal learning test and other procedures are described in the supplementary materials. Abbreviations: cog = cognitive, ROP = recent onset psychosis, ROD = recent onset depression, CHR = clinical high risk, rsfMRI = resting-state functional MRI, sMRI = structural MRI.


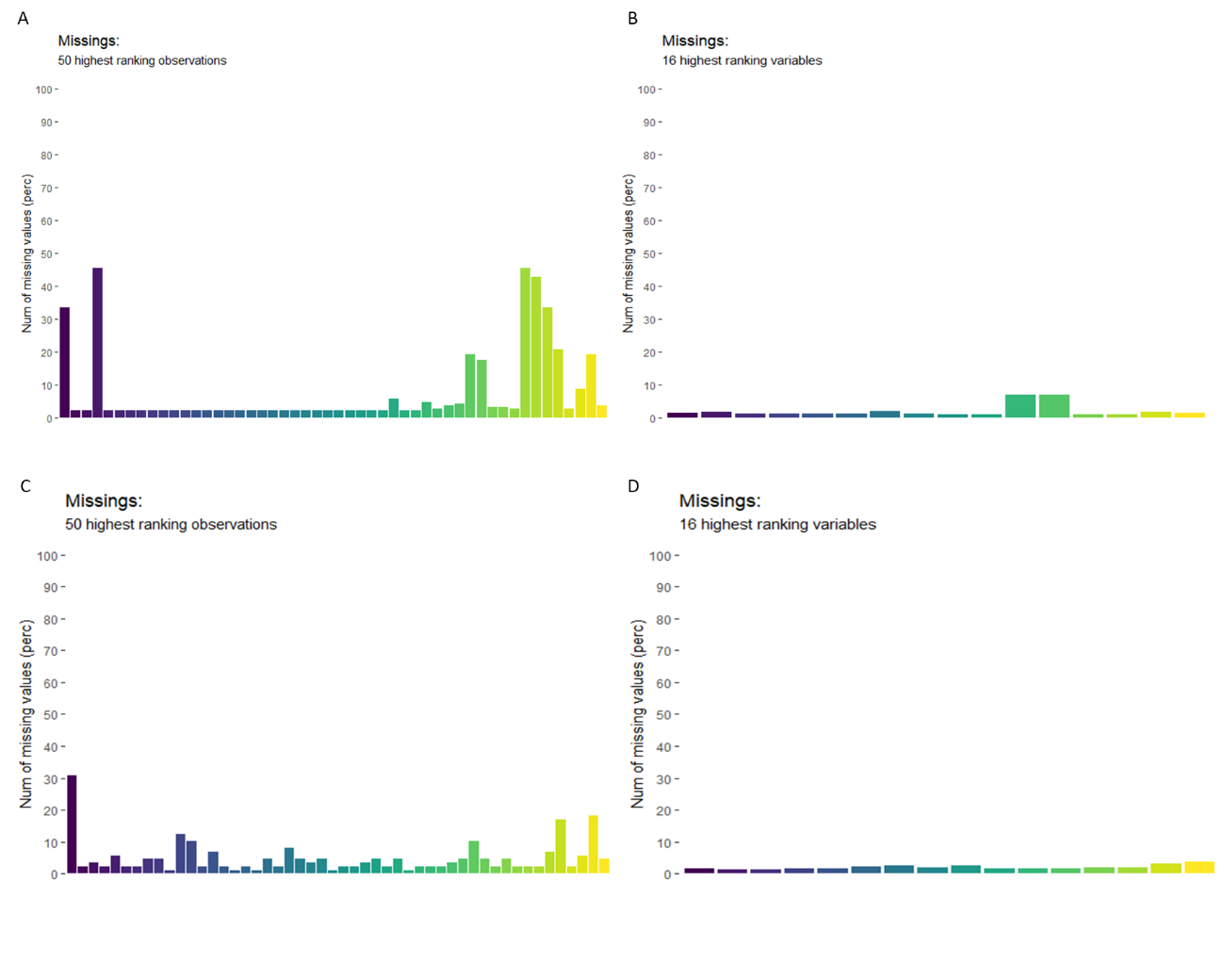


**Figure S2.** Missing values in neurocognitive data (all study groups together). Missing values in percentage are depicted for the discovery (upper panel) and replication sample (lower panel) per participant (A and C) and per cognitive variable (B and D) after exclusion of variables and participants (see preprocessing section in main manuscript and supplementary material).

**
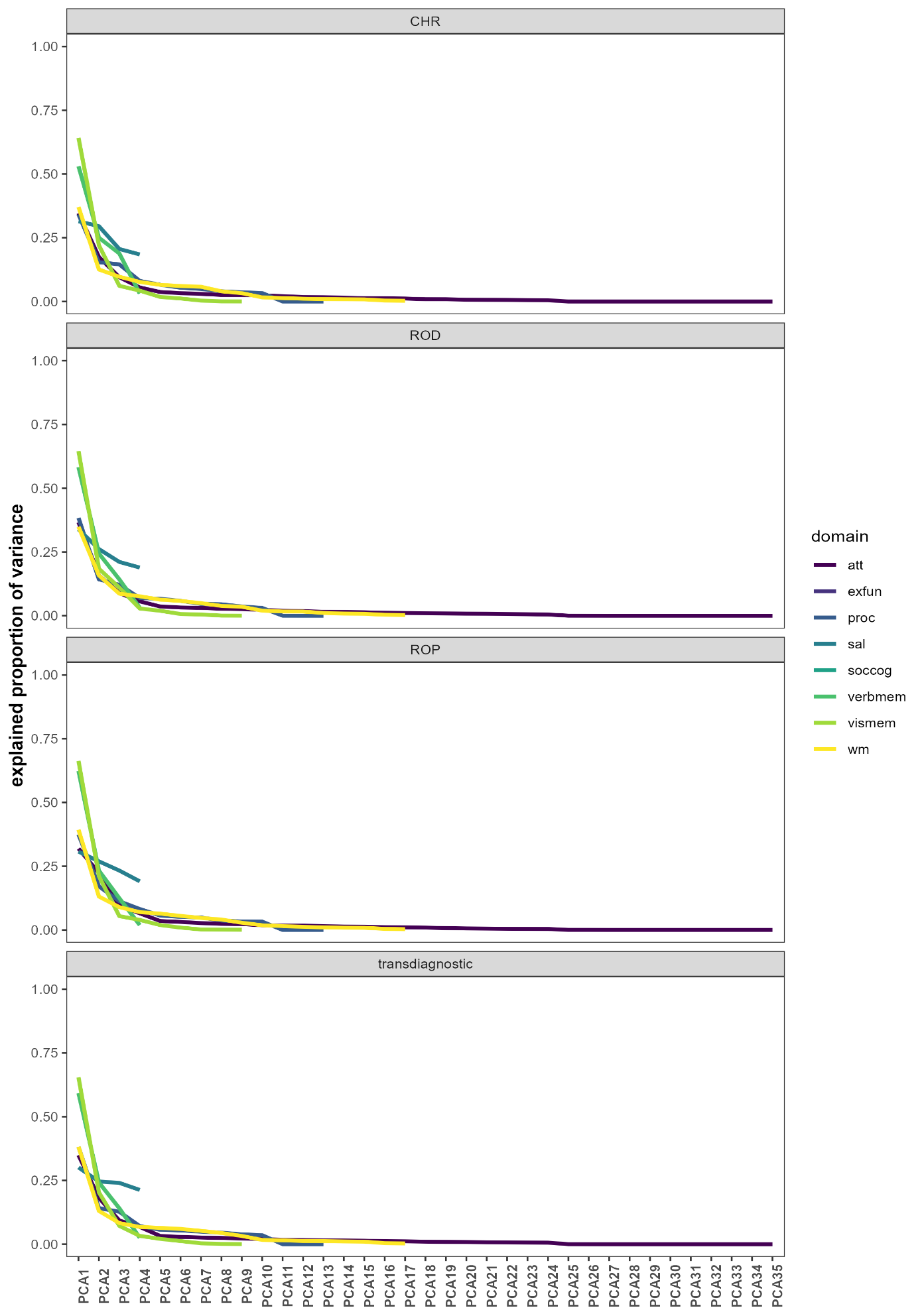
Figure S3.** Variance explanation of principal components (PC) for cognitive variables for the transdiagnostic sample and individual study groups. Cognitive variables were grouped according to their cognitive domain (different colors) and principal component analyses (PCA) were conducted separately. We selected only the first PC of each cognitive domain for cluster analysis. Abbreviations: att = attention; exfun = executive functioning, proc = processing speed, sal = salience, soccog = social cognition, verbmem = verbal memory, vismem = visual memory, wm = working memory.


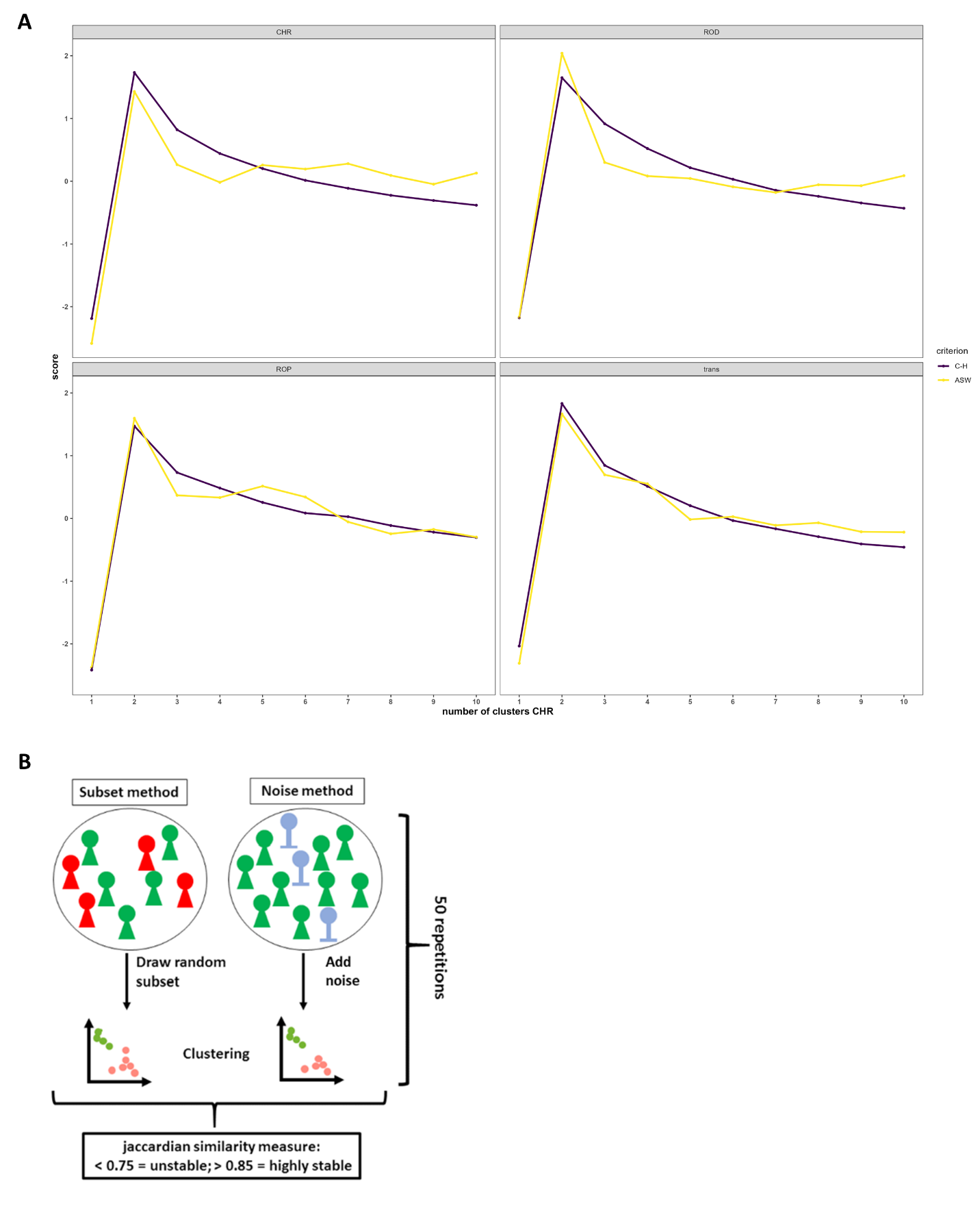


**Figure S4.** Selection of cluster number and cluster stability procedure. The number of clusters in the current analysis was determined by the average silhouette width (ASW) and the Calinski-Harabasz (C-H) index for each individual clustering based on the transdiagnostic sample (trans) and the samples including only psychosis patients (ROP), patients with depression (ROD, and individuals at clinical high-risk (CHR) (A). For both indices a maximum indicates the best ratio of between-cluster separation and within-cluster closeness for the selected cluster range (2-10). Cluster analysis was run on the 2-cluster-solution and its stability was assessed using a resampling approach (N_resample_ = 100) (B). Per iteration the ‘subset’ method subsampled a proportion of 50% of the data and the ‘noise’ method replaced 5% of the data with noise. For both methods we evaluated the proportion of cases clustered within the same group using Jaccard similarity.


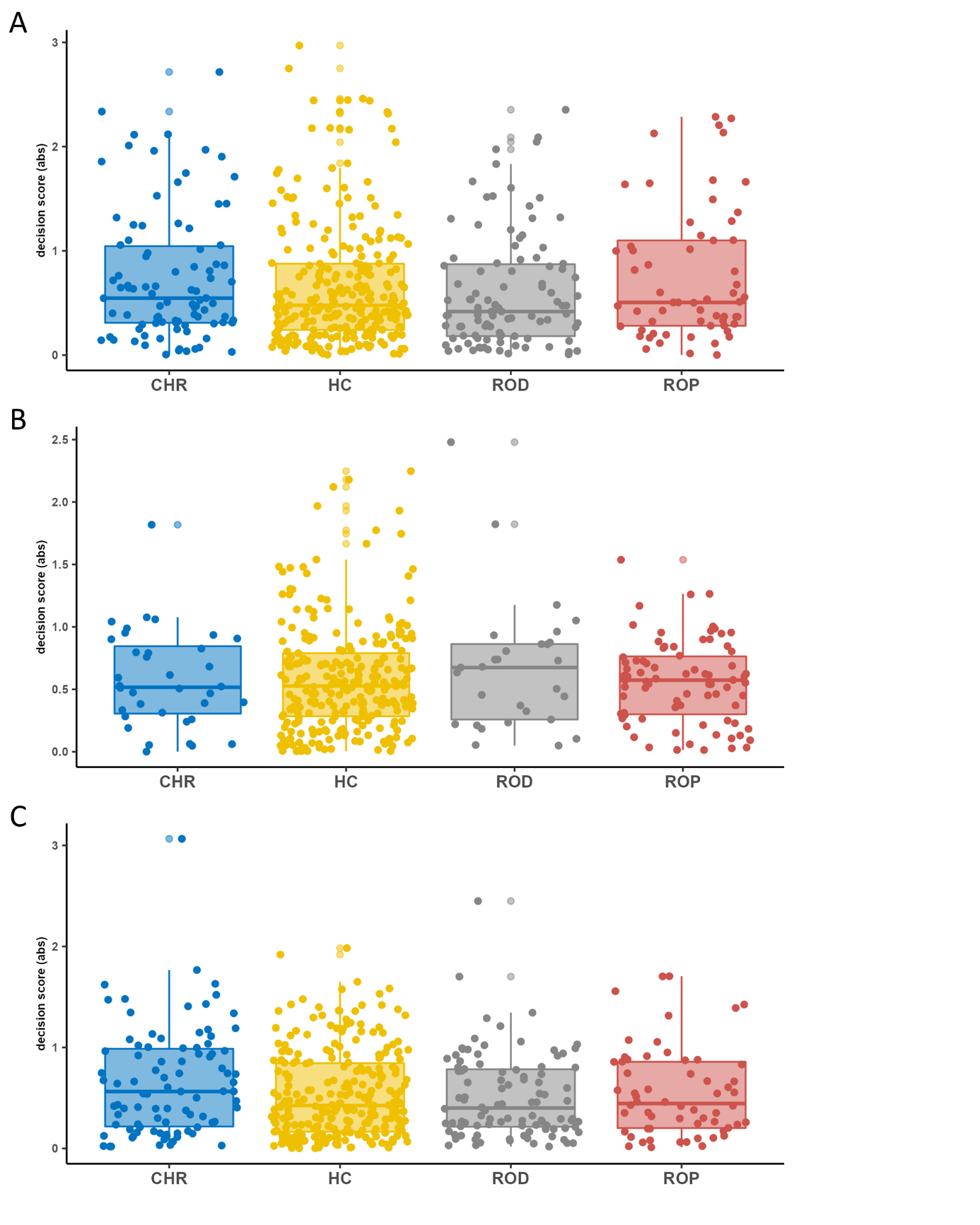


**Figure S5.** Support vector machine (SVM) decision scores between study groups in the three significant brain imaging models. Decision scores are absolute. Higher values indicate a higher certainty of the algorithm to classify an observation into one group or the other. (A) represents the decision scores for the classification between spared subgroup and HC based on sMRI. (B) and (C) represent the decision scores for the classification between impaired and HC, and spared and HC, respectively, based on rsfMRI. For all three classification models we obtained no significant differences across study groups (p > 0.12), indicating no classification benefit of a specific study group within a cluster. Abbreviations: ROP = recent-onset psychosis, ROD = recent-onset depression, CHR = clinical high-risk, HC = healthy controls.


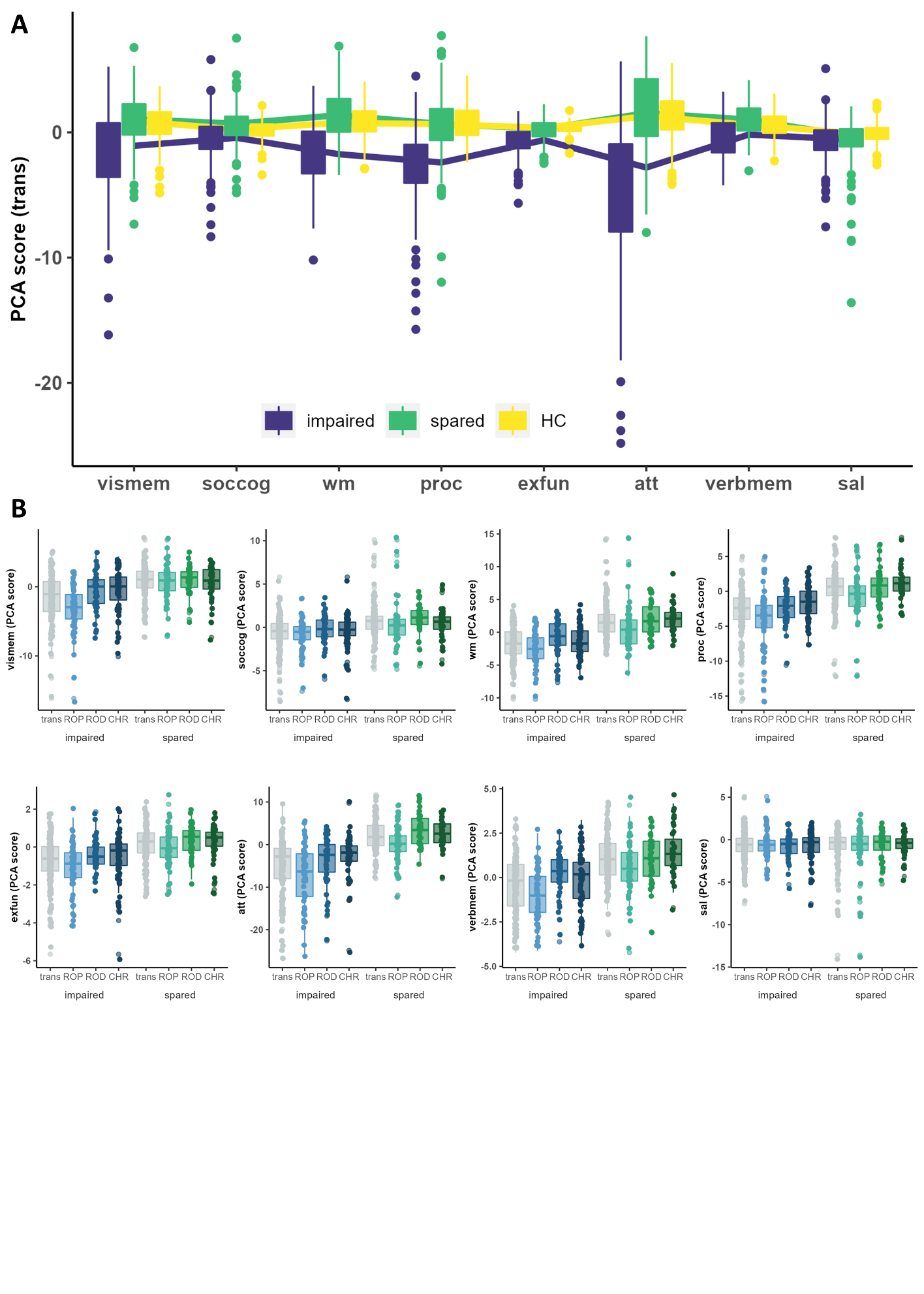


**Figure S6.** Cognitive characteristics of clusters based on the transdiagnostic and individual clustering analyses in the replication sample. Section A represents the cognitive performances of impaired (blue) and spared clusters (green) for the transdiagnostic cluster solution. Section B represents the cognitive performances of impaired (shades of blue) and spared (shades of green) clusters for the clusterings based on recent-onset depression patients (ROD), recent-onset psychosis patients (ROP) and clinical high-risk individuals (CHR) separately. For comparison impaired and spared clusters of the transdiagnostic cluster solution are shown in grey. For both sections: High principal component (PCA) scores represent high performance. Abbreviations: vismem = visual memory; soccog = social cognition; wm = working memory; proc = processing speed; exfun = executive functioning; att = attention; sal = salience; verbmem = verbal memory.


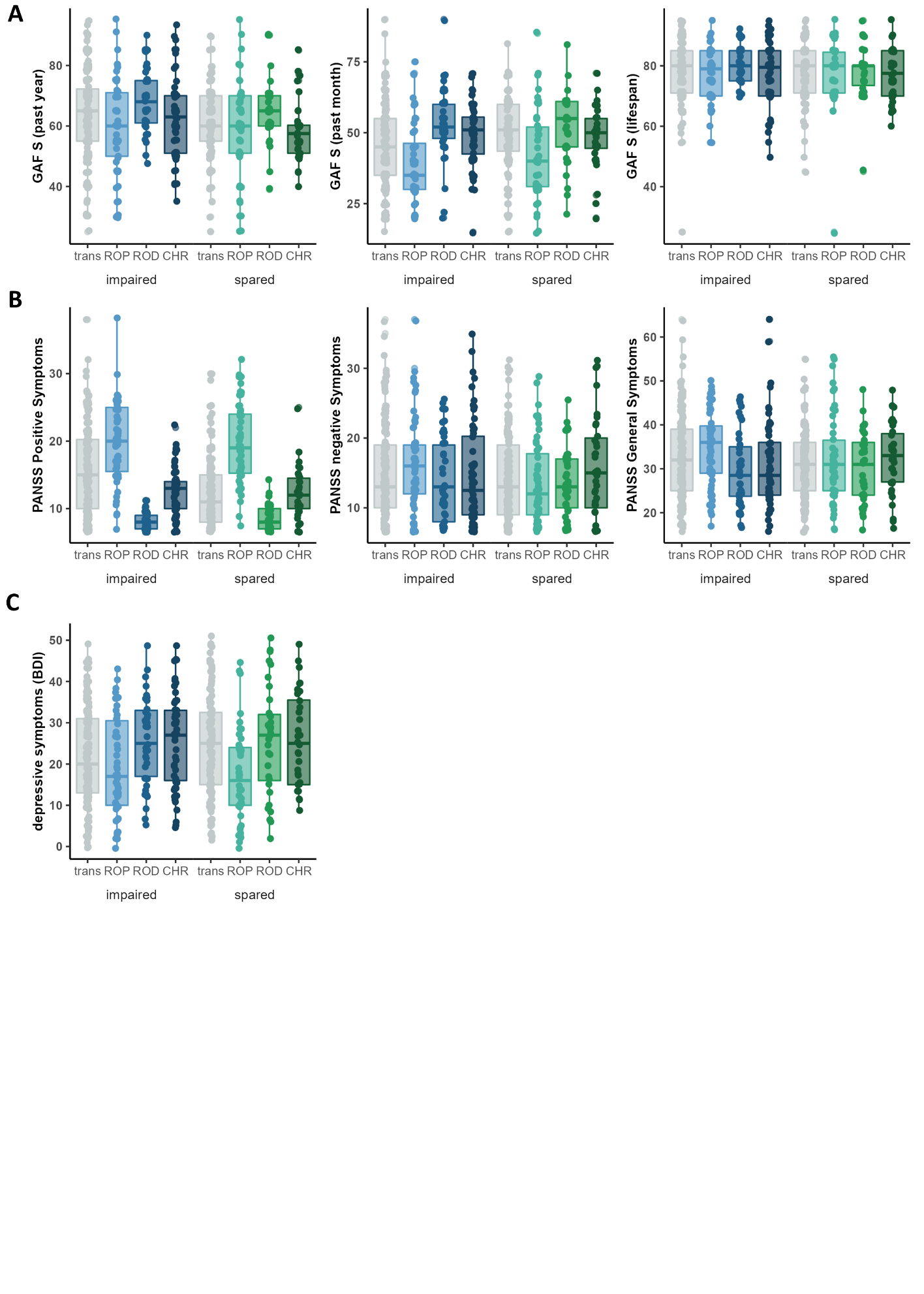


**Figure S7.** Functional characteristics and characteristics with respect to symptoms of the clusters based on the transdiagnostic and individual clustering analyses in the replication sample. Functioning differences (A) and psychotic (B) and depressive (C) symptom differences of impaired (shades of blue) and spared (shades of green) clusters for the clusterings based on recent-onset depression patients (ROD), recent-onset psychosis patients (ROP) and clinical high-risk individuals (CHR) separately. For comparison impaired and spared clusters of the transdiagnostic cluster solution are shown in grey. Abbreviations: GAF S = Global Assessment of Functioning (symptom scale); PANSS = Positive and Negative Syndrome Scale; BDI = Beck’s Depression Inventory.
